# Type I interferons drive MAIT cell functions against bacterial pneumonia

**DOI:** 10.1084/jem.20230037

**Published:** 2023-07-26

**Authors:** Juan Carlos López-Rodríguez, Steven J. Hancock, Kelin Li, Stefania Crotta, Christopher Barrington, Alejandro Suárez-Bonnet, Simon L. Priestnall, Jeffrey Aubé, Andreas Wack, Paul Klenerman, Jose A. Bengoechea, Patricia Barral

**Affiliations:** 1 The Peter Gorer Department of Immunobiology, King’s College London, London, UK; 2 The Francis Crick Institute, London, UK; 3 Wellcome-Wolfson Institute for Experimental Medicine. School of Medicine, Dentistry and Biomedical Sciences, Queen’s University Belfast, Belfast, UK; 4 Division of Chemical Biology and Medicinal Chemistry, UNC Eshelman School of Pharmacy, University of North Carolina at Chapel Hill, Chapel Hill, NC, USA; 5 Department of Pathobiology and Population Sciences, The Royal Veterinary College, Hatfield, UK; 6 Peter Medawar Building for Pathogen Research, Oxford, UK

## Abstract

Mucosal-associated invariant T (MAIT) cells are abundant in the lung and contribute to host defense against infections. During bacterial infections, MAIT cell activation has been proposed to require T cell receptor (TCR)–mediated recognition of antigens derived from the riboflavin synthesis pathway presented by the antigen-presenting molecule MR1. MAIT cells can also be activated by cytokines in an MR1-independent manner, yet the contribution of MR1-dependent vs. -independent signals to MAIT cell functions in vivo remains unclear. Here, we use *Klebsiella pneumoniae* as a model of bacterial pneumonia and demonstrate that MAIT cell activation is independent of MR1 and primarily driven by type I interferons (IFNs). During *Klebsiella* infection, type I IFNs stimulate activation of murine and human MAIT cells, induce a Th1/cytotoxic transcriptional program, and modulate MAIT cell location within the lungs. Consequently, adoptive transfer or boosting of pulmonary MAIT cells protect mice from *Klebsiella* infection, with protection being dependent on direct type I IFN signaling on MAIT cells. These findings reveal type I IFNs as new molecular targets to manipulate MAIT cell functions during bacterial infections.

## Teaser

This report identifies a central role for type I interferons in governing the activation and effector functions of murine and human MAIT cells during pulmonary infection with *Klebsiella pneumoniae*.

## Graphical Abstract

**Figure:**
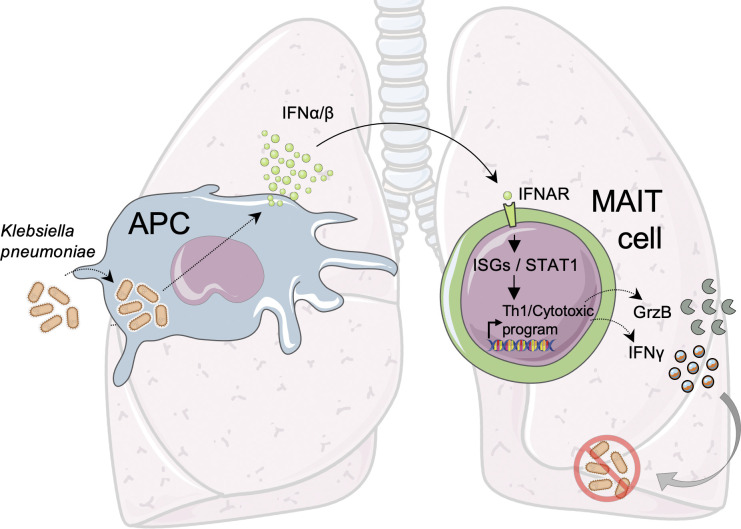

## Introduction

Unconventional T cells comprise several families of lymphocytes that rapidly sense the presence of microbes at mucosal and non-mucosal surfaces, functioning as sentinels of tissue integrity during infections. Among the families of unconventional T cells, mucosal-associated invariant T (MAIT) cells have recently emerged as central players of immunity in the airways, performing protective roles against bacterial and viral infections (Godfrey et al., 2019; Leeansyah et al., 2021; Provine and Klenerman, 2020; Ussher et al., 2018). MAIT cells carry a semi-invariant TCR that recognizes antigens presented by the ubiquitously expressed MHC class I–related molecule 1 (MR1; Kjer-Nielsen et al., 2012). Several bacteria and fungi produce antigens to stimulate MAIT cells, which include metabolites derived from the riboflavin synthesis pathway (Corbett et al., 2014). Consequently, MAIT cells are activated in vitro by many of these pathogens and contribute to protective immune responses in vivo against pulmonary infection by bacteria such as *Francisella tularensis* or *Legionella longbeachae* (Meierovics et al., 2013; Meierovics and Cowley, 2016; Wang et al., 2018, 2019). In addition to MR1-TCR–dependent activation, MAIT cells can also be activated in response to cytokines through MR1-independent mechanisms. This is the case during viral infections in which cytokines are sufficient to stimulate MAIT cells. For instance, during infection with dengue, hepatitis C, or influenza virus, IL-18 drives MAIT cell activation in synergy with IL-12, IL-15, and/or type I IFNs (van Wilgenburg et al., 2016). Despite the increasingly well-characterized functions of MAIT cells, the mechanisms driving their local activation and effector function in the tissues remain poorly defined. Moreover, whether MR1-independent signals are sufficient to control MAIT cell functions in vivo during bacterial infections has not yet been addressed.

Type I IFNs (including IFNα and IFNβ) are known for driving a potent immunomodulatory action combating viral infections, yet their functions during bacterial infection remain unclear (McNab et al., 2015). Type I IFNs have been shown to play a protective role in some bacterial infections (*Escherichia coli*, *Helicobacter pylori*), but they appear to be harmful in others (*Listeria monocytogenes*). The mechanisms by which IFNα/β promote host protection or susceptibility to bacterial pathogens are poorly defined and seem to be bacterium dependent (Kovarik et al., 2016). Thus, understanding the mechanisms by which type I IFNs contribute to the immune response against bacterial infections is of utmost importance. Type I IFNs are part of a complex crossregulatory network and can directly control the activation and function of a variety of immune and non-immune cells. IFNα/β can act directly on conventional CD4 and CD8 T cells and have stimulatory or inhibitory effects on T cell survival and proliferation, cytokine production, and memory formation (Crouse et al., 2015; Kuka et al., 2019). In the case of MAIT cells, type I IFNs contribute to MAIT cell activation during viral infection or adenovirus vector vaccine by acting in synergy with IL-18 (Provine et al., 2021; van Wilgenburg et al., 2016). Furthermore, in vitro experiments suggest that IFNα can synergize with TCR signals to increase MAIT cell effector functions (Lamichhane et al., 2020; Pavlovic et al., 2020). Nonetheless, whether type I IFNs modulate MAIT cell activation and/or functions during bacterial infections and the relevance of this regulation in vivo remain unknown.

*Klebsiella pneumoniae* is a Gram-negative opportunistic enterobacterium causing severe pneumonia, sepsis, and urinary tract infections (Bengoechea and Sa Pessoa, 2019). This bacterium has been cataloged as a global health threat due to the increasing prevalence of antibiotic-resistant isolates and has been identified by the World Health Organization as of critical priority for the development of new treatments (Theuretzbacher, 2017). Previous studies suggested that MAIT cells are activated by and contribute to the control of *K. pneumoniae* (Georgel et al., 2011; Le Bourhis et al., 2010), although the mechanisms underlying these processes remain unknown. *Klebsiella* encodes genes involved in riboflavin biosynthesis (Vitreschak et al., 2002), suggesting an MR1-dependent mechanism of MAIT cell activation. However, we unexpectedly found that *Klebsiella* drives activation and effector functions of murine and human MAIT cells through an MR1-independent mechanism. Accordingly, our experiments demonstrate that *Klebsiella* induces activation, tissue relocation, and a Th1/cytotoxic transcriptional program in MAIT cells, all of which are regulated by type I IFN, independently on MR1-TCR signals. Consequently, transfer of MAIT cells to immune-deficient mice or expansion of pulmonary MAIT cells protect mice from *Klebsiella* infection, with protection being dependent on direct type I IFN signaling on MAIT cells. Our data indicate that type I IFNs drive the activation and antimicrobial function of MAIT cells during bacterial pneumonia, contributing to host protection.

## Results and discussion

### MR1-independent activation of MAIT cells during *K. pneumoniae* infection

**Figure 1.**
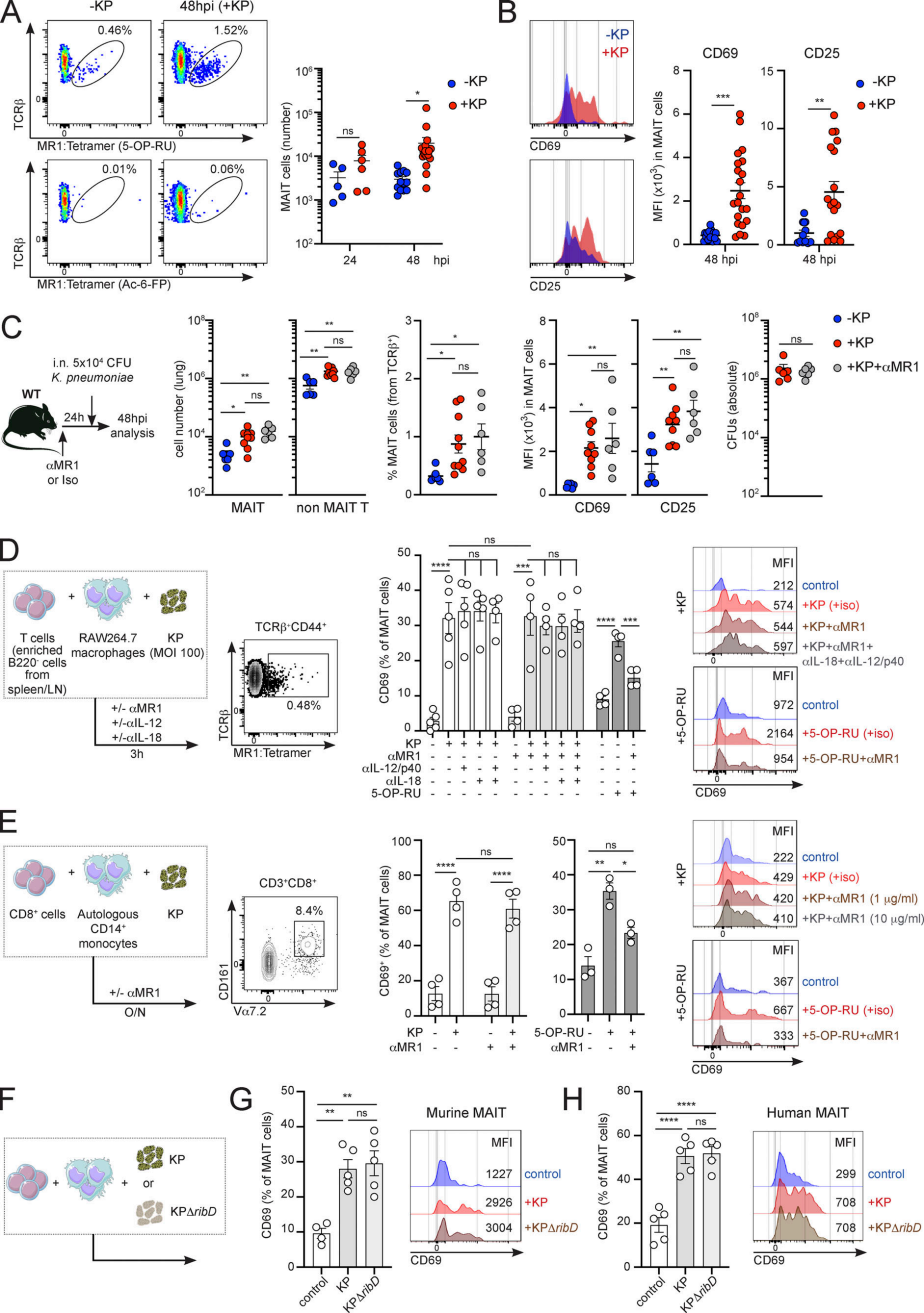
***K. pneumoniae* induces MR1-independent accumulation and activation of pulmonary MAIT cells. (A and B)** Flow cytometry plots and quantification of MAIT cell absolute numbers (A) or mean fluorescence intensity (MFI) for MAIT cells’ CD69 and CD25 (B) in the lungs of WT mice 48 h after infection with *K. pneumoniae* (+KP, red) or uninfected controls (−KP, blue). Stainings with 5-OP-RU MR1–loaded tetramer (top) or control Ac-6-FP–loaded tetramer (bottom) are shown in A. Lines represent mean ± SEM, each dot is a mouse (*n* = 5–17), and data are pooled from more than three independent experiments. *P < 0.05, **P < 0.01, ***P < 0.001; two-way ANOVA with Tukey’s multiple comparisons (A) or unpaired two-tailed *t* test (B). **(C)** WT mice were injected with αMR1 antibody or isotype control prior to *Klebsiella* infection. Absolute numbers and frequency of pulmonary MAIT cells and non-MAIT T cells, MFI for CD69 and CD25, and lung bacterial burden (CFUs) are shown. Lines represent mean ± SEM, each dot is a mouse (*n* = 6–9), and data are pooled from two to three independent experiments. *P < 0.05, **P < 0.01; one-way ANOVA with Tukey’s multiple comparisons. **(D)** Murine T cells (enriched from spleen and inguinal lymph nodes of WT mice as B220^−^ cells) were cultured (for 3 h) with RAW264.7 macrophages in the presence/absence of alive *K. pneumoniae* (MOI = 100) or 5-OP-RU and blocking antibodies as indicated. The frequency of CD69^+^ MAIT cells measured by flow cytometry is shown. Bars represent mean ± SEM, each dot is an independent experiment (*n* = 4–5) performed with T cells obtained from five to six mice. Right: Representative histograms for MAIT cells’ CD69 (including MFI values) after culture with *Klebsiella* (top) or 5-OP-RU (bottom). ***P < 0.001, ****P < 0.0001; ns, not significant, two-way ANOVA with Tukey’s multiple comparisons test. **(E)** Human CD8^+^ T cells were cultured (for 18 h) with autologous CD14^+^ monocytes in the presence/absence of (fixed) *K. pneumoniae* or 5-OP-RU and blocking antibodies as indicated. The frequency of CD69^+^ MAIT cells measured by flow cytometry is shown. Bars represent mean ± SEM, each dot is an independent experiment (*n* = 4) with data from two donors. Right: Representative histograms for MAIT cells’ CD69 (including MFI values) after culture with *Klebsiella* (top) or 5-OP-RU (bottom). *P < 0.5, **P < 0.01, ****P < 0.0001; ns, not significant, two-way ANOVA with Tukey’s multiple comparisons test. **(F)** Experimental setup for coculture of murine (G) and human (H) MAIT cells with WT (KP, Kp43816) or mutant (KPΔ*ribD*) *Klebsiella*. **(G)** Murine T cells (enriched from spleen and inguinal lymph nodes of WT mice as B220^−^ cells) were cultured (for 3 h) with RAW264.7 macrophages in the presence/absence of WT (KP, Kp43816) or mutant KPΔ*ribD* as indicated. Representative histograms and frequency of CD69^+^ MAIT cells measured by flow cytometry are shown. Bars represent mean ± SEM, data are pooled from two independent experiments performed with T cells obtained from three mice. **P < 0.01; ns, not significant, one-way ANOVA with Tukey’s multiple comparisons test. **(H)** Human CD8^+^ T cells were cultured (for 18 h) with autologous CD14^+^ monocytes in the presence/absence of WT (KP, Kp43816) or mutant KPΔ*ribD* as indicated. Representative histograms and frequency of CD69^+^ MAIT cells measured by flow cytometry are shown. Bars represent mean ± SEM, data pooled from two independent experiments. ****P < 0.0001; ns, not significant, one-way ANOVA with Tukey’s multiple comparisons test.

**Figure S1.**
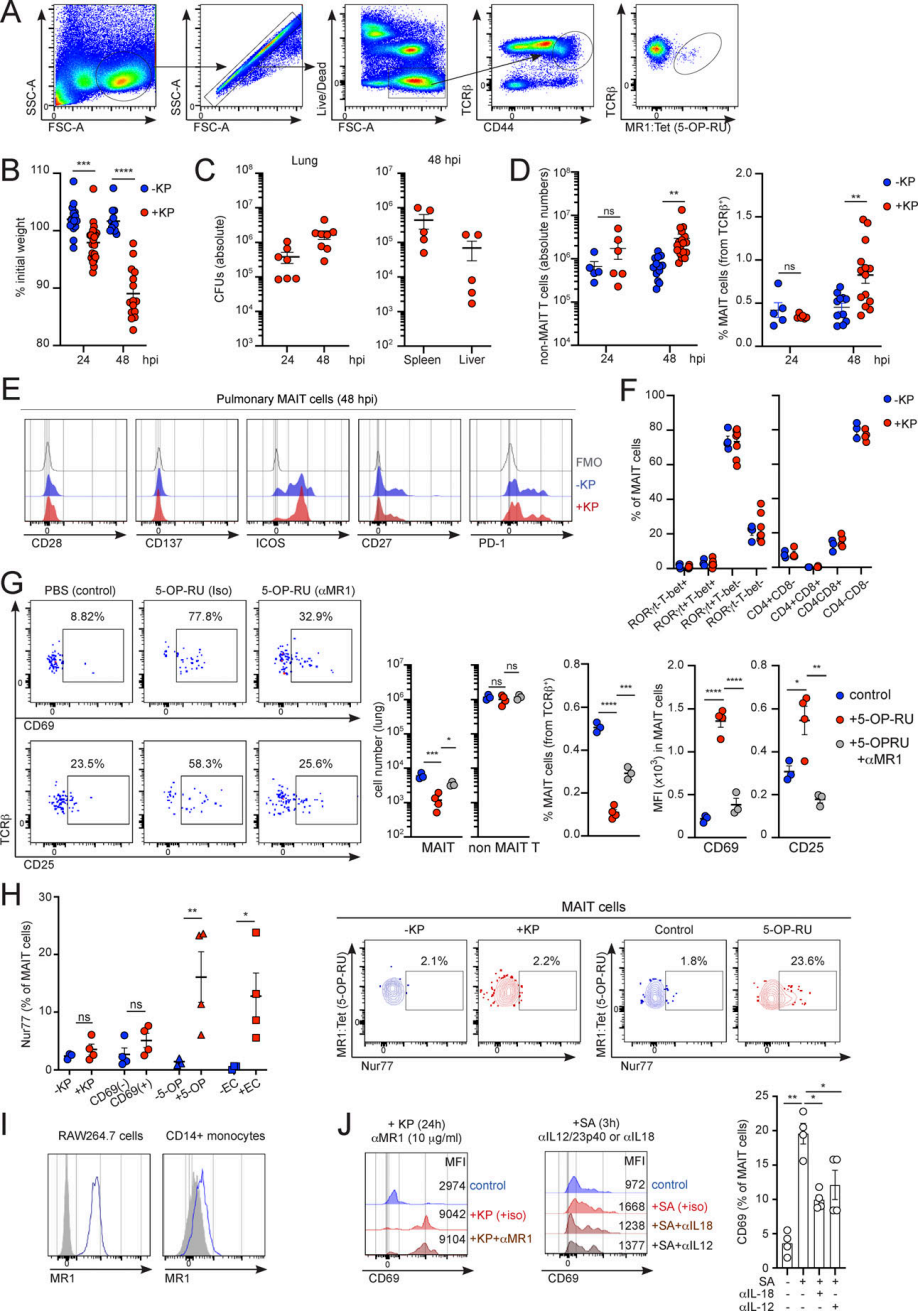
**Pulmonary MAIT cell populations during *K. pneumonia* infection. (A)** Flow cytometry gating strategy for pulmonary MAIT cells (Zombie^−^TCRβ^+^CD44^+^MR1:Tetramer (5-OP-RU)^+^) in WT C57BL/6J mice. **(B)** Body-weight loss in WT mice at 24 and 48 h after infection with *K. pneumoniae* (+KP, red) or uninfected controls (−KP, blue). Lines represent mean ± SEM, each dot is a mouse (*n* = 12–23). ***P < 0.001, ****P < 0.0001, two-way ANOVA with Tukey’s multiple comparisons test. **(C)** Bacterial burden in the lungs, spleen, and liver of infected WT mice at the indicated time points; each dot is a mouse (*n* = 5–8). **(D)** Absolute numbers of pulmonary non-MAIT T cells (left) and MAIT cell frequencies (respect to TCRβ^+^ cells, right) in WT mice 48 h after infection with *K. pneumoniae* (+KP, red) or uninfected controls (−KP, blue). Lines represent mean ± SEM, each dot is a mouse (*n* = 5–17), data pooled from more than three independent experiments. **P < 0.01; two-way ANOVA with Tukey’s multiple comparisons. **(E)** Representative flow cytometry plots showing expression of the depicted markers in pulmonary MAIT cells 48 h after infection with *K. pneumoniae.* FMO, fluorescence minus one. **(F)** Flow cytometry quantification of RORγt/T-bet expression and CD4/CD8 expression for pulmonary MAIT cells 48 h after infection with *K. pneumoniae* (+KP, red) or uninfected controls (−KP, blue). Each dot is a mouse (*n* = 3–7); data pooled from two to three independent experiments. **(G)** Flow cytometry plots showing expression of CD69 and CD25 in pulmonary MAIT cells from WT mice receiving i.n. 5-OP-RU or PBS (control). Mice were injected with anti-MR1 blocking antibody or isotype control (iso) 24 h earlier. Absolute numbers and frequency of pulmonary MAIT cells and non-MAIT T cells and MFI for CD69 and CD25 are shown. Lines represent mean ± SEM, each dot is a mouse (*n* = 3–4). *P < 0.05, **P < 0.01, ***P < 0.001, ****P < 0.0001; one-way ANOVA with Tukey’s multiple comparisons. **(H)** Left: Nur77 expression in pulmonary MAIT cells from WT mice infected with *Klebsiella* (left), CD69^+^ and CD69^−^ MAIT cells from *Klebsiella*-infected mice (middle), pulmonary MAIT cells from 5-OP-RU injected mice (5-OP), or splenic MAIT cells from mice infected with *E. coli* (i.p., 5 × 10^3^ CFUs/mouse, right). Lines represent mean ± SEM; each dot is a mouse (*n* = 4); data pooled from two to three independent experiments. Right: Representative flow cytometry plots for Nur77 expression for pulmonary MAIT cells for mice infected with *Klebsiella* (left) or receiving 5-OP-RU (right). *P < 0.05; **P < 0.01; ns, not significant, unpaired two-tailed *t* test. **(I)** Representative flow cytometry profiles showing expression of MR1 in RAW264.7 cells (left) or CD14^+^ monocytes (right). **(J)** Murine T cells (enriched from spleen and inguinal lymph nodes of WT mice as B220^−^ cells) were cultured with RAW264.7 macrophages in the presence/absence of *K. pneumoniae* (for 24 h, left) or *S*.* aureus* (SA, for 3 h, right) and blocking antibodies as indicated. Representative histograms for MAIT cells CD69 (including MFI values) and frequency of CD69^+^ MAIT cells in response to SA (right) are shown. Bars represent mean ± SEM, data are pooled from two independent experiments with T cell isolated from three to five mice. *P < 0.5, **P < 0.01, ANOVA with Tukey’s multiple comparisons test.

While MAIT cells have been proposed to participate in the immune response against *K. pneumoniae* (Georgel et al., 2011; Le Bourhis et al., 2010), the signals driving MAIT cell activation in the tissues remain unknown. To investigate the mechanisms controlling the activation of pulmonary MAIT cells, we infected WT mice with *K. pneumoniae* (KP, strain Kp43816) through intranasal (i.n.) challenge with 5 × 10^4^ live bacteria (Fig. 1 and Fig. S1). This strain is widely used to study the host response to *Klebsiella* as it recapitulates *Klebsiella*-triggered human pneumonia, inducing acute disease with fatal systemic spread at a low infectious dose (Chen et al., 2016a; Lawlor et al., 2005). Infected mice rapidly lost weight, and we recovered bacteria from the lungs of inoculated animals 24 h post-infection (hpi) and from distal organs (spleen and liver) at 48 hpi (Fig. S1, B and C). Phenotyping of pulmonary MAIT cells (defined as CD44^+^TCRβ^+^MR1(5-OP-RU)-Tetramer^+^; Fig. 1, A and B; and Fig. S1 A) indicated that these cells were readily activated in response to *Klebsiella* as we detected upregulation of the activation markers CD69 and CD25 (Fig. 1, A and B). MAIT cell activation was accompanied by an increase in MAIT cell frequencies and absolute numbers at 48 hpi (Fig. 1, A and B; and Fig. S1 D). We further phenotyped pulmonary MAIT cells at 48 hpi by examining the expression of transcription factors (T-bet and RORγt) and surface markers (Fig. S1, E and F). As previously described (Rahimpour et al., 2015), the majority of MAIT cells in the lungs of uninfected animals are T-bet^−^RORγt^+^ and lack expression of CD4 and CD8, and these proportions are not altered in response to *Klebsiella* infection (Fig. S1 F).

Since the *K. pneumoniae* genome contains genes encoding enzymes of the riboflavin pathway (Vitreschak et al., 2002), we investigated whether pulmonary MAIT cell activation was mediated by MR1-TCR engagement by injecting mice with αMR1 blocking antibody prior to infection (Fig. 1 C). Surprisingly, MR1 blocking did not affect MAIT cell activation, and MAIT cell absolute numbers (and frequencies), upregulation of CD69 and CD25, and lung bacterial burden (CFUs) were comparable for infected mice receiving αMR1 or isotype control (Fig. 1 C). Importantly, the same dose of αMR1 reduced activation markers in pulmonary MAIT cells after i.n. inoculation of mice with the MAIT cell ligand (5-(2-oxopropylideneamino)-6-D-ribitylaminouracil; 5-OP-RU), confirming its effectiveness (Fig. S1 G). Moreover, endogenous MAIT cell Nur77 (which is used as an indicator of TCR signaling; Moran et al., 2011) was not significantly upregulated in pulmonary MAIT cells in response to *Klebsiella* infection (vs. uninfected controls) or when comparing CD69^+^ vs. CD69^−^ MAIT cells within infected animals (Fig. S1 H). However, we detected Nur77 upregulation in pulmonary MAIT cells after i.n. challenge with 5-OP-RU as well as in splenic cells after infection of mice with *E. coli* (Fig. S1 H). Thus, these data suggest that pulmonary MAIT cells are activated during *K. pneumoniae* infection thorough an MR1-independent mechanism.

Activation of MAIT cells during bacterial infections has been proposed to require TCR engagement, although during viral infections cytokines (such as IL-12 and IL-18) function in a TCR-independent manner to stimulate MAIT cells (van Wilgenburg et al., 2016). To investigate the signals controlling MAIT cell activation in response to *Klebsiella,* we cocultured in vitro murine T cells (enriched as B220^−^ cells from spleen and lymph nodes) with RAW264.7 macrophages (which constitutively express surface MR1) in the presence/absence of *Klebsiella* for 3 h (Fig. 1 D and Fig. S1, I and J). As expected, *Klebsiella* induced the activation of MAIT cells (gated as CD44^+^TCRβ^+^MR1(5-OP-RU)-Tetramer^+^), as evidenced by CD69 upregulation, yet CD69 levels were unaffected by αMR1 blockade (1 μg/ml, Fig. 1 D). Moreover, blockade of other known MAIT cell-activating cytokines (IL-18, IL-12/23p40) alone, or in combination with αMR1, did not alter CD69 levels (Fig. 1 D). Similarly, MAIT cell CD69 was also unaltered when cells were cocultured with *Klebsiella* for a longer period of time (24 h) in the presence of a higher dose of αMR1 (10 μg/ml; Fig. S1 J). On the other hand, αMR1 blocked activation of MAIT cells in the presence of 5-OP-RU (Fig. 1 D, right), while αIL-18 or αIL-12/23p40 reduced MAIT cell CD69 in response to *Staphylococcus aureus* (SA, Fig. S1 J), confirming the functionality of these antibodies in our experimental settings. Comparable results were obtained while measuring activation of human MAIT cells in response to *Klebsiella* (Fig. 1 E). Coculture of human circulating CD8^+^ T cells (MAIT cells gated as CD3^+^CD8^+^CD161^+^Vα7.2^+^ cells) with autologous CD14^+^ monocytes in the presence of *Klebsiella* resulted in MAIT cell CD69 upregulation, which was independent of MR1 (1 μg/ml; Fig. 1 E). MAIT cell activation in response to *Klebsiella* was also unaltered by a higher dose of αMR1 (10 μg/ml), while this antibody efficiently blocked human MAIT cell CD69 upregulation in response to 5-OP-RU (Fig. 1 E, right).

To unequivocally address the requirement for microbial riboflavin metabolites in driving MAIT cell responses during *Klebsiella* infection, we constructed a *K. pneumoniae* mutant strain in which we deleted the *ribD* gene (KPΔ*ribD*; Fig. 1, F–H), which is essential for the production of 5-A-RU, the precursor of the MAIT cell antigens 5-OP-RU and 5-OE-RU (Corbett et al., 2014). We cocultured in vitro murine T cells (enriched as B220^−^ cells from spleen and lymph nodes) with RAW264.7 macrophages in the presence of WT (KP, Kp43816) or KPΔ*ribD* *Klebsiella* for 3 h (Fig. 1 G). Strikingly, both WT and KPΔ*ribD* induced activation of MAIT cells to comparable levels as evidenced by CD69 upregulation (Fig. 1 G). Comparable results were obtained for human MAIT cells which when cultured with Kp43816 or the *ribD* mutant showed similar levels of CD69 induction (Fig. 1 H).

Collectively, these data suggest that *K. pneumoniae* mediates activation of murine and human MAIT cells primarily through an MR1-independent mechanism.

### *Klebsiella* induces a dominant type I IFN signature in MAIT cells

**Figure 2.**
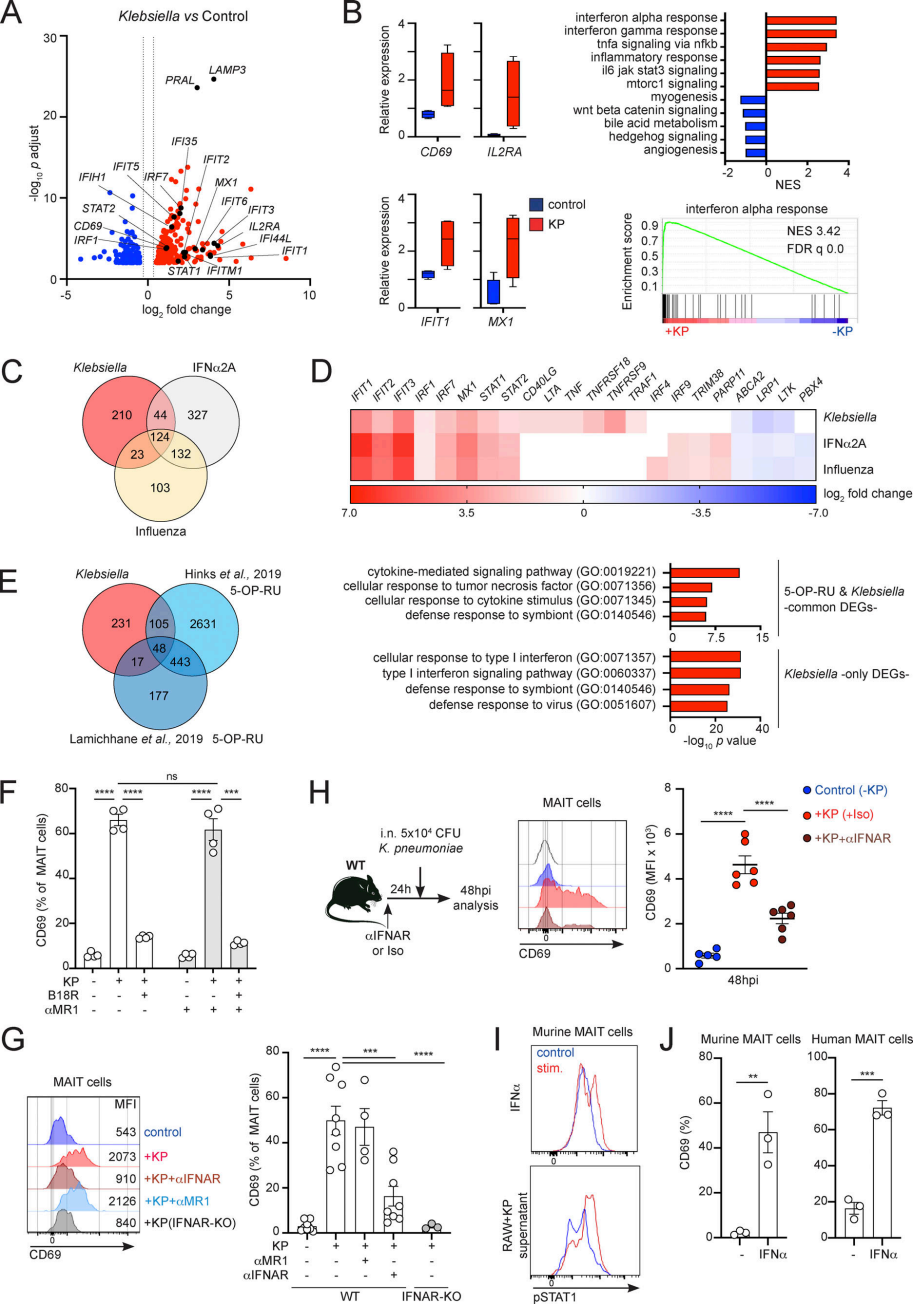
**Type I IFN drives MAIT cell activation in response to *Klebsiella*. (A–E)** Human MAIT cells were sorted from PBMCs (four healthy donors) after incubation with *Klebsiella*, IFNα2A, influenza virus, or no stimuli (control) and subjected to RNAseq analyses. **(A)** Volcano plot including DEGs up- (red) or downregulated (blue) in response to *Klebsiella* vs. control. Labeled genes are colored black. A log_2_ fold change cut-off of 0.5 and adjusted P value cut-off of 0.01 were applied. **(B)** Left: Relative gene expression of selected transcripts in MAIT cells exposed to *Klebsiella* (red) vs. control (blue). Boxes show 25th to 75th percentiles with whiskers being max/min values. Top right: Results of GSEA hallmark pathway analysis showing top enriched gene sets. Normalized enrichment score (NES) values indicate enrichment (red bars, positive NES) in response to *Klebsiella* or in control (blue bars, negative NES). Bottom right: GSEA enrichment plot of “interferon alpha response” gene set. **(C)** Venn diagram showing the number of DEGs in MAIT cells in response to *Klebsiella*, IFNα2A, or influenza. **(D)** Heatmap for fold change of selected transcripts significantly changed in MAIT cells in response to *Klebsiella*, IFNα2A, or influenza vs. control. **(E)** Venn diagram (left) showing the number of DEGs in MAIT cells in response to *Klebsiella* and 5-OP-RU (transcriptomic data obtained from Hinks et al. [2019]; Lamichhane et al. [2019]). Functional enrichment analysis (right) of genes positively changed on MAIT cells exposed to 5-OP-RU and *Klebsiella* (“common DEGs,” top) or for DEGs enriched only on MAIT cells exposed to *Klebsiella* but not in cells treated with 5-OP-RU (“*Klebsiella* only DEGs,” bottom). Top GO terms are shown ranked by P values. **(F)** Human CD8^+^ T cells were cultured (for 18 h) with autologous CD14^+^ monocytes in presence/absence of (fixed) *K. pneumoniae* and blocking antibodies and/or the type I IFN inhibitor B18R as indicated. Frequency of CD69^+^ MAIT cells (measured by flow cytometry) is shown. Bars represent mean ± SEM, each dot is an independent experiment (*n* = 4) with data from two donors, ***P < 0.001, ****P < 0.0001; ns, not significant, two-way ANOVA with Tukey’s multiple comparisons test. **(G)** Murine MAIT cells were sorted from the lungs of WT or IFNAR-KO mice (previously injected with 5-OP-RU+LPS to expand the MAIT cell population) and cultured (for 18 h) with RAW264.7 macrophages in the presence/absence of (fixed) *K. pneumoniae* and blocking antibodies as indicated. Left: Representative histograms for MAIT cells’ CD69 (including MFI values) after culture with *Klebsiella* and blocking antibodies. Right: Frequency of CD69^+^ MAIT cells measured by flow cytometry. Bars represent mean ± SEM, each dot is an independent experiment (*n* = 3–8) performed with MAIT cells obtained from 5 to 10 mice. ***P < 0.001, ****P < 0.0001, one-way ANOVA with Tukey’s multiple comparisons test. **(H)** WT mice were injected with αIFNAR antibody or isotype control prior to infection with *K. pneumoniae*. Flow cytometry plot for CD69 expression (middle) and quantification of CD69 MFI (right) for pulmonary MAIT cells are shown. Lines represent mean ± SEM, each dot is a mouse (*n* = 5–6), and data are pooled from two independent experiments. ****P < 0.0001, one-way ANOVA with Tukey’s multiple comparisons test. **(I)** Murine MAIT cells were sorted from the lungs of WT mice (previously injected with 5-OP-RU+LPS to expand the MAIT cell population) and cultured with IFNα (top) or the supernatant of *Klebsiella*-treated RAW264.7 cells (bottom). Representative flow cytometry plots (from three independent experiments) show phosphorylation of STAT1 in MAIT cells after 60 min of stimulation. Red profile = stimulated cells; blue = unstimulated controls. **(J)** MAIT cells were sort-purified from murine lungs (left) or human PBMCs (right) and incubated with IFNα for 18 h. Frequency of CD69^+^ MAIT cells (measured by flow cytometry) is shown. Bars represent mean ± SEM, and each dot is a replicate from three independent experiments performed with MAIT cells obtained from five mice each (left) or pooled data from two donors (right). **P < 0.01, ***P < 0.001, unpaired two-tailed *t* test.

**Figure S2.**
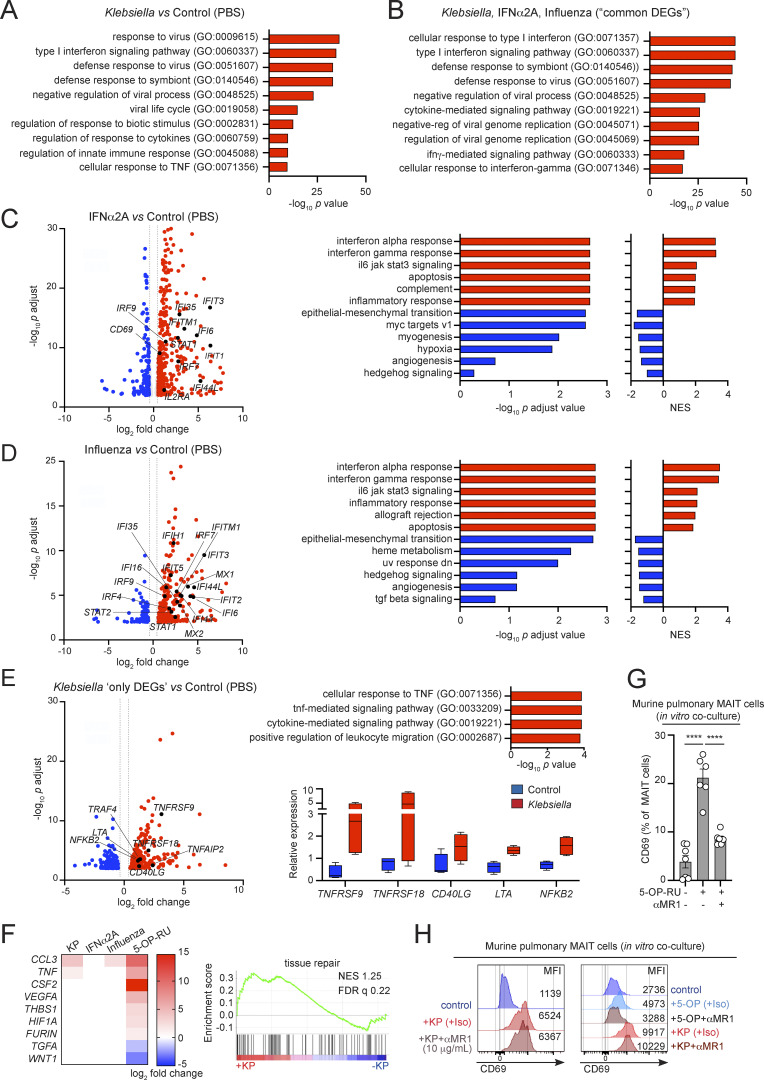
**MAIT cells’ transcriptional programs. (A–F)** Human MAIT cells were sorted from PBMCs (four healthy donors) after incubation with *Klebsiella*, IFNα2A, influenza virus, or no stimuli (control) and subjected to RNAseq analyses. **(A and B)** Functional enrichment analysis of genes positively changed in MAIT cells exposed to *Klebsiella* vs. control (A), or for DEGs enriched in MAIT cells exposed to *Klebsiella*, IFNα, and influenza (B; “common DEGs”). The GO terms are shown ranked by P values. Enrichment and P values (from a Fisher’s exact test with Bonferroni correction) were calculated with PANTHER tools. **(C and D)** Human MAIT cells were sorted from PBMCs (four healthy donors) after incubation with IFNα2A (C), influenza virus (D; or no stimuli [control]) and subjected to RNAseq analyses. Left: Volcano plots including DEGs up- (red) or downregulated (blue) in response to IFNα vs. control (C) or influenza vs. control (D). Labeled genes are colored black. A log_2_ fold change cut-off of 0.5 and adjusted P value cut-off of 0.01 were applied. Right: Results of GSEA hallmark pathway analysis showing enriched gene sets. Adjusted P values and NES for the indicated pathways are shown (red bars, positive NES; blue bars, negative NES). **(E)** Analyses of DEGs enriched in MAIT cells exposed to *Klebsiella* but not in cells exposed to IFNα or influenza (*K. pneumoniae* “only DEGs”). Labeled genes are colored black. A log_2_ fold change cut-off of 0.5 and adjusted P value cut off of 0.01 were applied. Top right: Functional enrichment analysis of *K. pneumoniae* “only DEGs.” The GO terms are shown ranked by P values. Enrichment and P values (from a Fisher’s exact test with Bonferroni correction) were calculated with PANTHER tools. Bottom right: Relative gene expression of selected transcripts in MAIT cells exposed to *Klebsiella* (red) vs. control (blue). Boxes show 25th to 75th percentiles with whiskers being max/min values. **(F)** Heatmap (left) and GSEA plot (right) for “tissue repair” associated genes as described by Hinks et al. (2019). **(G and H)** MAIT cells were sorted from the lungs of WT mice (previously injected with 5-OP-RU+LPS to expand the MAIT cell population) and cultured (18 h) with RAW264.7 macrophages in the presence/absence of *K. pneumoniae*, 5-OP-RU, and/or anti-MR1 blocking antibody (1 or 10 μg/ml) or isotype (control) as indicated. Frequencies (G) and representative histograms (H) for MAIT cells CD69 (including MFI values) are shown from two independent experiments performed with MAIT cells obtained from three to five mice. Bars represent mean ± SEM, ****P < 0.0001, one-way ANOVA with Tukey’s multiple comparisons test.

Next, we investigated the signaling pathways driving activation of MAIT cells in response to *Klebsiella*. To obtain an unbiased overview of the phenotype and properties of MAIT cells in response to infection, we isolated human MAIT cells (from peripheral blood mononuclear cells [PBMCs] from four healthy donors) after overnight culture in the presence/absence of *Klebsiella* and analyzed their gene-expression profile by RNA sequencing (RNAseq). These analyses revealed 401 genes that were differentially expressed (DEG) in MAIT cells ± *Klebsiella* (adjusted P value <0.01, log_2_ fold change >0.5), with 255 upregulated and 146 downregulated genes (Fig. 2 and Fig. S2). As expected, MAIT cells were markedly activated by *Klebsiella* as analyses of genes involved in early T cell activation confirmed the upregulation of transcripts such as *CD69* or *IL2RA* (*CD25*; Fig. 2, A and B; and Fig. S2). Gene set enrichment analyses (GSEA) showed enrichment of an IFNα response signature induced in MAIT cells in response to *Klebsiella* (*IRF1*, *IRF7*, *IFIT1*, and *MX1*; Fig. 2 B). In line with this, gene ontology (GO) analysis using PANTHER analysis tools demonstrated that *Klebsiella* induced a significant enrichment for genes related to “type I IFN signaling pathway” as well as “response to virus” (Fig. S2 A). Thus, *Klebsiella* induces a dominant type I IFN signature in MAIT cells.

To further investigate the effect of type I IFNs in MAIT cell activation, we compared the transcriptional program acquired by MAIT cells in response to *Klebsiella* with that of cells stimulated with IFNα or influenza virus (Fig. 2, C and D; and Fig. S2, B–E). Venn diagrams highlight the overlapping and unique transcriptional signatures elicited by these three stimulations (Fig. 2 C). Strikingly, from the 401 DEG induced by *Klebsiella*, more than 40% of genes (168) were also induced after stimulation with IFNα and 124 genes were common for the three stimulations. As expected, most of these common genes are IFN-related genes, and GO enrichment analysis revealed a dominant type I IFN signaling signature (Fig. S2 B). On the other hand, 210 genes appeared exclusively altered by *K. pneumoniae*, being substantially enriched in genes involved in “cellular response to TNF” including those related to TNF-dependent signaling (*CD137* [*TNFRSF9*], *CSF1*, and *TNFAIP2*) and members of the TNF superfamily (*CD40L* [*TNFSF5*], *GITR* [*TNFRSF18*], and *LTA* [*TNFB*]; Fig. 2 D and Fig. S2 E).

We next compared the transcriptional program induced in MAIT cells by *Klebsiella*, with two publicly available datasets of MAIT cells stimulated with the MAIT cell antigen 5-OP-RU (Hinks et al., 2019; Lamichhane et al., 2019; Fig. 2 E). We detected 170 DEGs that were induced by *Klebsiella* and also found to be altered in response to 5-OP-RU in one or both datasets. GO analyses of these “common” DEGs showed an enrichment on “cytokine-mediated signaling” pathways, while DEGs only induced by *Klebsiella* but not 5-OP-RU (231) retained enrichment in IFN signatures. Also, while MAIT cells have been shown to acquire a “tissue-repair” signature in response to TCR stimulation (Hinks et al., 2019; Lamichhane et al., 2019; Leng et al., 2019), we didn’t detect a significant enrichment of tissue repair–related genes in response to *Klebsiella* (Fig. S2 F).

Given the dominant type I IFN signature induced by *Klebsiella*, we investigated whether type I IFNs are the main drivers controlling MAIT cell responses during infection. Indeed, in in vitro cocultures using human (Fig. 2 F) or pulmonary murine (Fig. 2 G) cells, blocking type I IFN signaling with the IFNα/β inhibitor B18R (human) or IFNα/β receptor (IFNAR) blocking antibody (αIFNAR, mouse) reduced CD69 upregulation in MAIT cells in response to *Klebsiella*. This process was independent of MR1 as it was not blocked by αMR1 incubation, while this antibody efficiently blocked 5-OP-RU–mediated CD69 upregulation in murine pulmonary MAIT cells (Fig. S2, G and H). Similar results were obtained in cocultures performed with RAW264.7 macrophages and MAIT cells isolated from mice lacking type I IFN receptor (IFNAR-KO), which show strongly impaired *Klebsiella*-mediated CD69 upregulation (Fig. 2 G, right), indicating a cell-intrinsic effect for type I IFN signaling in regulating MAIT cell responses. Moreover, type I IFNs also controlled MAIT cell-CD69 upregulation in vivo (Fig. 2 H). Blocking IFNAR in vivo by injection of a blocking antibody to WT mice prior to *Klebsiella* infection also resulted in significantly lower CD69 levels vs. isotype-treated mice (Fig. 2 H).

**Figure S3.**
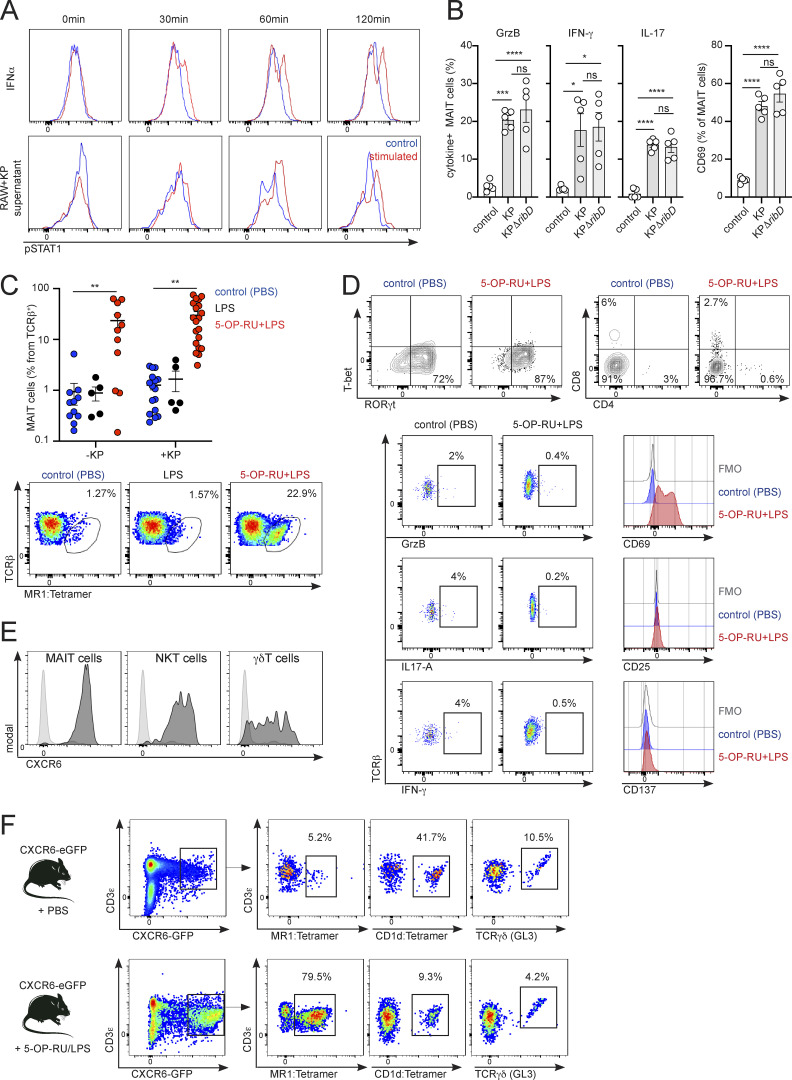
**Type I IFN–dependent MAIT cell activation. (A)** Murine MAIT cells were sorted from the lungs of WT mice (previously injected with 5-OP-RU+LPS to expand the MAIT cell population) and cultured with IFNα or the supernatant of *Klebsiella*-treated RAW264.7 cells. Representative flow cytometry plots (from three independent experiments) show phosphorylation of STAT1 in MAIT cells at the indicated time points after stimulation with IFNα (top) or the supernatant of *Klebsiella*-treated RAW264.7 cells (bottom). Red profile = stimulated cells; blue = unstimulated controls. **(B)** MAIT cells were sorted from the lungs of WT mice (previously injected with 5-OP-RU+LPS to expand the MAIT cell population) and cultured (for 18 h) with RAW264.7 macrophages in the presence/absence of WT (KP, Kp43816) or mutant KPΔ*ribD* as indicated. Frequencies of Granzyme B (GrzB^+^), IFN-γ^+^, IL-17^+^, or CD69^+^ MAIT cells is shown; data are pooled from two independent experiments with MAIT cells obtained from three to five mice. Bars represent mean ± SEM, *P < 0.5, ***P < 0.001, ****P < 0.0001; ns, not significant, one-way ANOVA with Tukey’s multiple comparisons test. **(C)** Flow cytometry plots and quantification of MAIT cell frequency in WT mice after i.n. challenges with PBS (blue), LPS (black), and 5-OP-RU+LPS (red). Lines represent mean ± SEM, each dot represents a mouse (*n* = 5–20). **P < 0.01, one-way ANOVA with Tukey’s multiple comparisons test. **(D)** Flow cytometry plots showing T-bet/RORγt, CD4/CD8, CD69/CD25/CD137, IFNγ/IL-17A/GramB in pulmonary MAIT cells from PBS-treated (control) vs. 5-OP-RU/LPS-treated mice. **(E)** Flow cytometry plots for CXCR6 expression in MAIT cells (CD3ε^+^MR1(5-OP-RU)Tetramer^+^), invariant natural killer T (iNKT) cells (CD3ε^+^CD1d(PBS-57)Tetramer^+^), and γδT cells (CD3ε^+^TCRγδ^+^) in the lungs of WT mice. **(F)** Flow cytometry plots showing CD3^+^GFP^+^ cells and frequency of MAIT cells, iNKT cells, and γδ T cells within the CD3^+^GFP^+^ population in the lung of CXCR6-eGFP mice without (top) or with (bottom) 5-OP-RU+LPS administration.

Next, we investigated the cell-intrinsic effects of type I IFNs on MAIT cells. Signal transducer and activator of transcription 1 (STAT1) is a critical component of IFN-I signaling, regulating the expression of hundreds of IFN-regulated genes, and it is transcriptionally induced in MAIT cells in response to *Klebsiella* or IFNα (Fig. 2 D). Thus, we analyzed the phosphorylation of STAT1 in sorted murine pulmonary MAIT cells in response to incubation with IFNα or with the supernatant of *Klebsiella*-infected RAW264.7 macrophages. Both type I IFN and RAW-supernatant induced a time-dependent phosphorylation of STAT1 in MAIT cells, which was evident already at 15–30 min of stimulation and sustained after 2 h (Fig. 2 I and Fig. S3 A). In line with this, incubation of sort-purified MAIT cells with IFNα was sufficient to induce CD69 upregulation of both murine and human MAIT cells (Fig. 2 J).

Altogether, these data demonstrate that *Klebsiella* induces a dominant type I IFN signature in MAIT cells, and type I IFNs are key drivers of MAIT cell responses during infection. Nonetheless, we cannot discard that other signals may also contribute to MAIT cell activation during *Klebsiella* infection. For example, RNAseq analyses detected a signature for “cellular response to TNF” induced by *Klebsiella* (Fig. 2 B), and TNF has been shown to contribute to MAIT cell activation in response to adenovirus-based vaccines (Provine et al., 2021).

### Intrinsic type I IFN signaling controls MAIT cell effector functions

**Figure 3.**
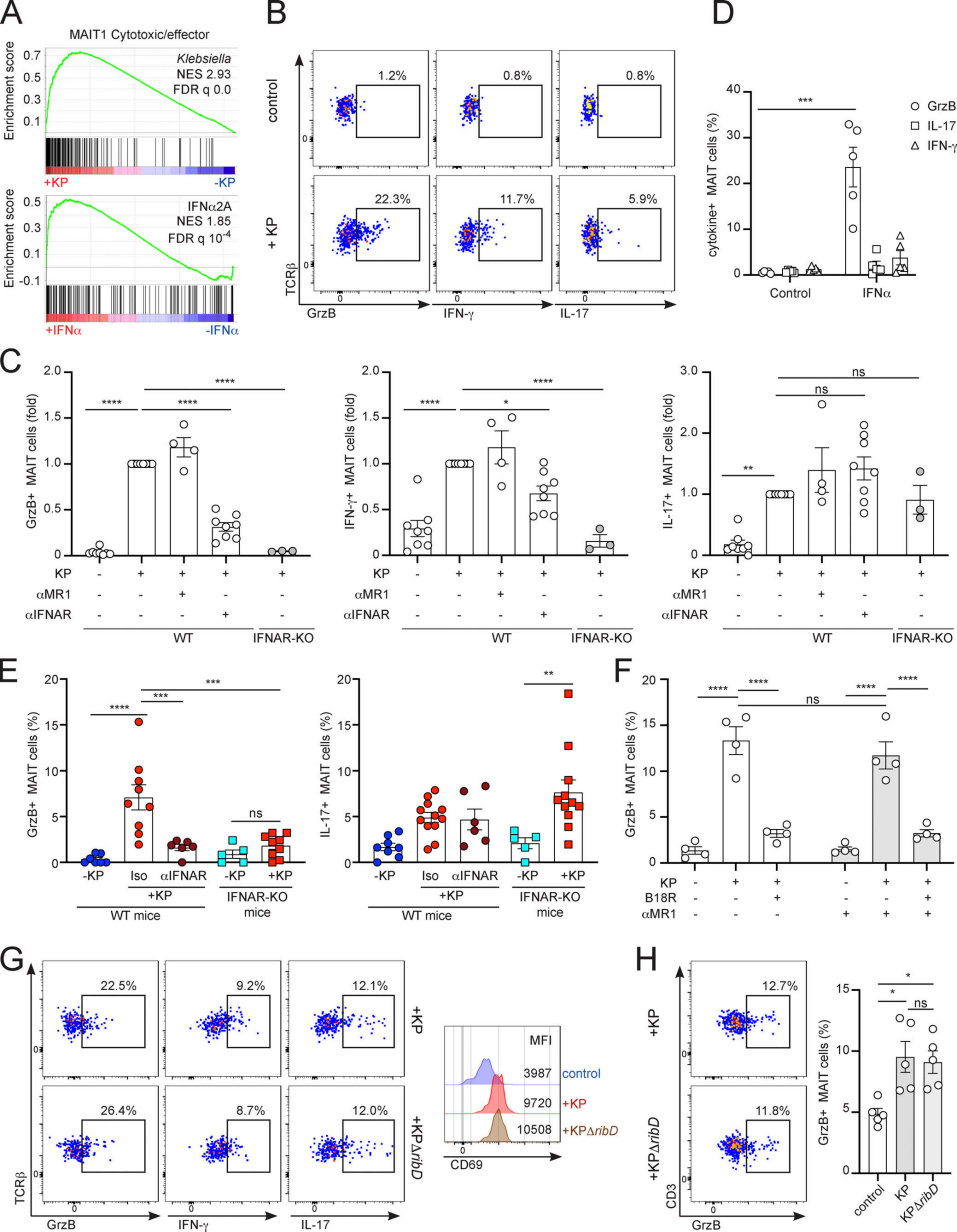
**Type I IFNs control MAIT cell effector functions during *K. pneumoniae* infection. (A)** GSEA plots showing enrichment for the transcriptional signatures for MAIT1-cytotoxic/effector phenotype (Vorkas et al., 2022) for MAIT cells treated with *Klebsiella* (top) or IFNα2A (bottom). FDR, false discovery rate. **(B and C)** Murine MAIT cells were sorted from the lungs of WT or IFNAR-KO mice (previously injected with 5-OP-RU+LPS to expand the MAIT cell population) and cultured (for 18 h) with RAW264.7 macrophages in the presence/absence of *K. pneumoniae* and blocking antibodies as indicated. Flow cytometry profiles (B) and normalized frequencies (respect to KP+ condition, C) of Granzyme B (GrzB)^+^, IFN-γ^+^, or IL-17^+^ MAIT cells (measured by flow cytometry) are shown. Bars represent mean ± SEM, each dot is an independent experiment (*n* = 3–8) performed with MAIT cells obtained from 5 to 10 mice. ns, not significant; *P < 0.05, **P < 0.01, ****P < 0.0001, one-way ANOVA with Tukey’s multiple comparisons test. **(D)** Secretion of Granzyme B (GrzB), IL-17A, and IFN-γ by sorted pulmonary murine MAIT cells after overnight incubation with IFNα (or untreated control). Bars represent mean ± SEM, data are pooled from five independent experiments performed with MAIT cells obtained from 5 to 10 mice each, ***P < 0.001; one-way ANOVA with Tukey’s multiple comparisons test. **(E)** WT mice or IFNAR-KO mice were infected with *Klebsiella* (+KP) and frequency of Granzyme B^+^ or IL-17^+^ MAIT cells in the lung of infected animals was measured at 48 hpi. WT mice were injected with αIFNAR antibody or isotype control (Iso) prior to infection as indicated. Bars represent mean ± SEM, each dot is a mouse (*n* = 5–12), and data pooled from three to four independent experiments. **P < 0.01 ***P < 0.001, ****P < 0.0001, one-way ANOVA with Tukey’s multiple comparisons test. **(F)** Human CD8^+^ T cells were cultured (for 18 h) with autologous CD14^+^ monocytes in the presence/absence of (fixed) *K. pneumoniae* and blocking antibodies and/or the type I IFN inhibitor B18R as indicated. The frequency of Granzyme B^+^ MAIT cells is shown. Bars represent mean ± SEM, each dot is an independent experiment (*n* = 4) with data from two donors, ****P < 0.0001; ns, not significant, two-way ANOVA with Tukey’s multiple comparisons test. **(G)** Murine MAIT cells were sorted from the lungs of WT mice (previously injected with 5-OP-RU+LPS to expand the MAIT cell population) and cultured (for 18 h) with RAW264.7 macrophages in presence of WT (KP, Kp43816) or mutant KPΔ*ribD* as indicated. Representative flow cytometry profiles of Granzyme B (GrzB)^+^, IFN-γ^+^, or IL-17^+^ MAIT cells (left) and CD69 expression (MFI, right) are shown. Data are representative from two independent experiments performed with MAIT cells obtained from three to five mice. **(H)** Human CD8^+^ T cells were cultured (for 18 h) with autologous CD14^+^ monocytes in presence/absence of WT (KP, Kp43816) or mutant KPΔ*ribD* as indicated. Flow cytometry profiles and frequencies of Granzyme B (GrzB)^+^ MAIT cells is shown. Bars represent mean ± SEM, with data pooled from two independent experiments. *P < 0.05; one-way ANOVA with Tukey’s multiple comparisons test.

Next, we investigated the MAIT cell effector functions induced by *Klebsiella* and the mechanisms regulating those functions. A recent single-cell transcriptomic atlas of human peripheral blood MAIT cells revealed their phenotypical and functional heterogeneity covering a broad range of homeostatic, effector, helper, tissue-infiltrating, regulatory, and exhausted phenotypes (Vorkas et al., 2022). Comparison of the transcriptomic signatures of MAIT cells activated with *Klebsiella* or IFNα revealed that these stimuli induced a significant enrichment in MAIT1-cytotoxic/effector signature in MAIT cells (Fig. 3 A), which included cells with a combined granzyme and Th1-helper associated phenotype (Vorkas et al., 2022).

To investigate the type I IFN–dependent effector functions of MAIT cells, we analyzed the production of effector molecules by culturing sorted murine pulmonary MAIT cells with RAW264.7 macrophages in the presence/absence of *Klebsiella* (Fig. 3, B and C). In response to *Klebsiella*, MAIT cells produced IFN-γ, Granzyme B, and to a lesser extent IL-17A (Fig. 3 B). Secretion of these three effectors was independent of MR1 (not affected by αMR1 antibody), yet secretion of Granzyme B and IFN-γ (but not IL-17) were regulated by type I IFNs. Accordingly, Granzyme B and IFN-γ secretion induced by *Klebsiella* was significantly reduced by an αIFNAR blocking antibody, as well as in cocultures performed with IFNAR-KO MAIT cells (Fig. 3 C). This suggests that intrinsic type I IFN signaling controls MAIT cell effector functions. In line with this, IFNα is sufficient to induce Granzyme B production by sort-purified pulmonary murine MAIT cells (Fig. 3 D). We also measured IFN-dependent secretion of cytokines by pulmonary MAIT cells in vivo after infection with *Klebsiella* (Fig. 3 E). MAIT cell–Granzyme B secretion was reduced when WT mice were injected with αIFNAR blocking antibody prior to infection as well as in infected IFNAR-KO mice, while IL-17 production was independent of IFNAR (Fig. 3 E). Comparable results were obtained for human MAIT cells when CD8^+^T cells were cocultured with autologous monocytes, as secretion of effector molecules was blocked by incubation with B18R (Fig. 3 F). Importantly, mutant KPΔ*ribD* induced secretion of cytokines (and CD69 upregulation) by murine (Fig. 3 G and Fig. S3 B) and human (Fig. 3 H) MAIT cells at comparable levels with those induced by Kp43816 (KP), further supporting MR1-independent mechanisms as main drivers of MAIT cell effector functions in response to *Klebsiella*. Collectively, these data indicate that type I IFNs are key regulators of MAIT cells during *Klebsiella* infection. The type I IFN effects are—at least in part—due to intrinsic IFN signaling on MAIT cells as it directly contributes to controlling their activation and effector functions. Thus, in response to *Klebsiella*, MAIT cells become potent effectors by sensing inflammation independently of cognate antigen.

### Type I IFNs regulate MAIT cell–dependent control of pulmonary *Klebsiella*

**Figure 4.**
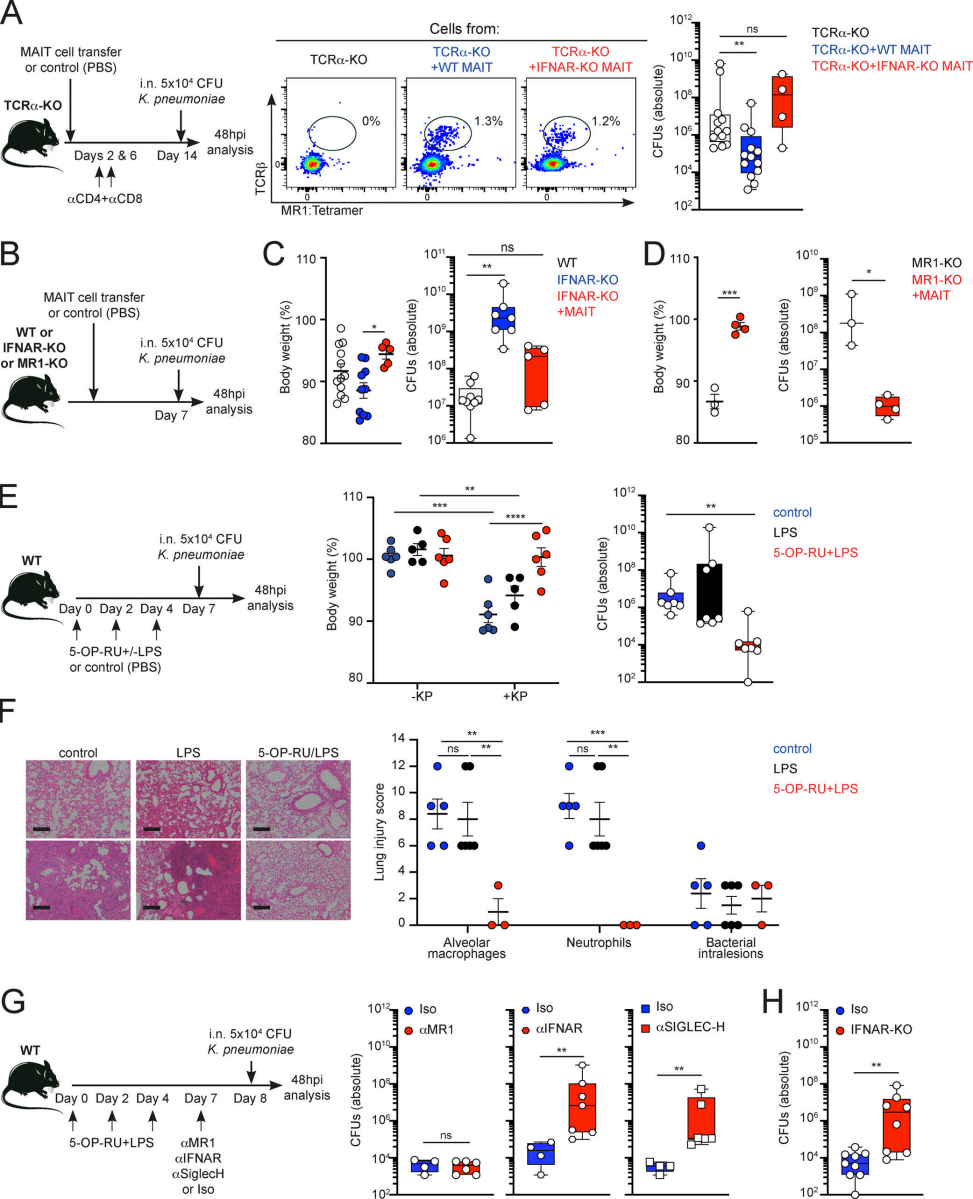
**Type I IFN controls MAIT cell–dependent protection from *K. pneumoniae* infection. (A)** Pulmonary MAIT cells from WT or IFNAR-KO mice (previously injected with 5-OP-RU+LPS to expand the MAIT cell population) were sorted and transferred intravenously into TCRα-deficient mice (day 0), followed by i.p. anti-CD4 and anti-CD8 antibody injection (days 2 and 6) to deplete any residual conventional T cells. On day 14, mice were infected with *Klebsiella*. Flow cytometry plots show the population of MAIT cells recovered from the lungs of control mice (no adoptive transfer) or those receiving WT (middle) or IFNAR-KO MAIT cells (right). Lung bacterial burden (absolute CFUs) were quantified at 48 hpi (right). Boxes show 25th to 75th percentiles with whiskers being max/min values, each dot is a mouse (*n* = 4–13), and data are pooled from three to six independent experiments, **P < 0.01, Kruskal–Wallis test. **(B–D)** Pulmonary MAIT cells from WT mice (previously injected with 5-OP-RU+LPS to expand the MAIT cell population) were sorted and transferred intravenously into IFNAR-KO (C) or MR1-KO mice (D). On day 7, after transfer, mice were infected with *Klebsiella*. Body-weight loss and lung bacterial burden (as absolute number of CFUs) were quantified at 48 hpi in WT, IFNAR-KO, or IFNAR-KO+MAIT mice (C) or in MR1-KO and MR1-KO+MAIT mice (D). Lines represent mean ± SEM (left) and boxes (right) show 25th to 75th percentiles with whiskers being max/min values, each dot is a mouse (*n* = 3–10), and data are pooled from two to four independent experiments, *P < 0.05, **P < 0.01, ***P < 0.001; ANOVA with Tukey’s multiple comparisons test (C, left), Kruskal–Wallis test (C, right), unpaired *t* test (D, left), Mann–Whitney test (D, right). **(E and F)** WT mice received three doses of 5-OP-RU+LPS, LPS, or PBS (control) as indicated (days 0, 2, and 4) and were infected with *Klebsiella* at day 7. **(E)** Body-weight loss (left) and lung bacterial burden (as absolute number of CFUs, right) are shown for mice receiving PBS (control, blue), LPS (black), or 5-OP-RU+LPS (red). Lines represent mean ± SEM (left) and boxes (right) show 25th to 75th percentiles with whiskers being max/min values. Each dot is a mouse (*n* = 6–7) and data are pooled from three to four independent experiments, **P < 0.01, ***P < 0.001, ****P < 0.0001 two-way ANOVA with Tukey’s multiple comparisons test (left) or Kruskal–Wallis test (right). **(F)** Lung sections stained with H&E of *Klebsiella*-infected WT mice pretreated with PBS, LPS, or 5-OP-RU+LPS as indicated. Quantification of lung injury (alveolar macrophages and neutrophils infiltration as well as intravascular bacterial lesions) is shown. Lung injury scores were determined by blinded scoring (from 0 to 12). Scale bar = 200 μm. Lines represent mean ± SEM, each dot is a mouse (*n* = 3–6), **P < 0.01, ***P < 0.001, two-way ANOVA with Tukey’s multiple comparisons test. **(G)** WT mice received three doses of 5-OP-RU+LPS and were injected with the depicted blocking antibodies (or isotype controls) prior to infection with *Klebsiella*. Bacterial burden (CFUs) recovered from the lungs are shown. Boxes show 25th to 75th percentiles with whiskers being max/min values, each dot is a mouse (*n* = 4–7), and data are pooled from two to three independent experiments, **P < 0.01, ns, not significant, Mann–Whitney test. **(H)** WT or IFNAR-KO mice were i.n. treated with 5-OP-RU+LPS and then infected with 5 × 10^4^ CFUs of *K. pneumoniae*. Plots represent lung bacterial burden (CFUs) at 48 hpi for WT (blue) and IFNAR-KO (red) mice. Boxes show 25th to 75th percentiles with whiskers being max/min values, each dot is a mouse (*n* = 7–8), and data are pooled from three independent experiments, **P < 0.01, Mann–Whitney test.

While our experiments confirm a key role for type I IFNs in regulating MAIT cell functions, the relevance of MAIT cell–intrinsic IFNAR for host protection remains unknown. To address whether IFNAR expression by MAIT cells confers protection against *Klebsiella,* we combined a variety of approaches to restrict IFNAR expression/depletion to MAIT cells in vivo (Fig. 4, A–C). First, we adapted a previously published approach (van Wilgenburg et al., 2018; Wang et al., 2018) by adoptively transferring pulmonary WT or IFNAR-KO MAIT cells into TCRα-KO mice (Fig. 4 A). Residual contaminating conventional T cells were depleted in recipients by injection of αCD4 and αCD8 antibodies. Mice were rested for 2 wk, to allow for MAIT cell expansion, and subsequently infected with *Klebsiella*. Strikingly, adoptively transferred MAIT cells contributed to the control of *Klebsiella* infection, as we recovered significantly lower CFUs from the lungs of mice receiving WT MAIT cells vs. control (untreated) animals (Fig. 4 A). However, the protective effect of MAIT cells was abrogated when the transferred cells were deficient in IFNAR, indicating that the protective role of MAIT cells is dependent on intrinsic IFNAR signaling (Fig. 4 A). As type I IFN signaling-deficient mice are susceptible to *K. pneumoniae* infection (Ivin et al., 2017), we asked whether MAIT cells are sufficient to control bacterial infection in an IFNAR-deficient context. To investigate this, we adoptively transferred WT pulmonary MAIT cells into IFNAR-KO recipients prior to infection with *Klebsiella* (Fig. 4, B and C). As previously described, IFNAR-KO mice showed increased bacterial loads in the lungs after infection in comparison with WT controls (Fig. 4 C). Strikingly, transfer of WT MAIT cells into IFNAR-KO recipients was sufficient to reduce bacterial loads (Fig. 4 C), confirming their protective role during *Klebsiella* infection. On the other hand, transfer of MAIT cells into MR1-KO recipients also conferred protection as evidenced by reduced body-weight loss and bacterial burden after infection, suggesting an MR1-independent mechanism driving MAIT cell protection in this setting (Fig. 4 D). Thus, these data indicate that type I IFN–mediated activation of MAIT cells drives their protective role during *K. pneumoniae* infection.

While MAIT cells are abundant in humans, in specific pathogen–free C57BL/6 mice, baseline frequencies of MAIT cells are very low. However, pulmonary MAIT cells can be expanded in mice via vaccination with 5-OP-RU in the presence of TLR agonists (Chen et al., 2016b; Hinks et al., 2019). Thus, we investigated whether MAIT cells contribute to the control of pulmonary *Klebsiella* infection and the relevance of type I IFNs in this system. To test this, we i.n. administered WT mice with 5-OP-RU+LPS and infected mice with *K. pneumoniae* a week later. As controls, we injected mice with either vehicle (PBS) or LPS (Fig. 4 E and Fig. S3 C). Administration of 5-OP-RU+LPS induced a strong increase in the total number of MAIT cells accumulated in the lung, which was not observed in mice receiving LPS alone or vehicle control (Fig. S3 C). MAIT cells from 5-OP-RU+LPS-treated mice showed comparable levels of cytokines, transcription factor expression, and surface markers, but increased levels of CD69 in comparison to MAIT cells from control (PBS treated) mice (Fig. S3 D). Strikingly, MAIT cell expansion resulted in protection from *Klebsiella* infection as evidenced by prevention of weight loss, decrease in bacterial CFUs, and decreased lung inflammation in 5-OP-RU+LPS-treated mice (Fig. 4, E and F). This protection was not observed in mice treated exclusively with LPS, which showed comparable numbers of MAIT cells, weight loss, bacterial CFUs, and lung pathology as control (untreated) mice (Fig. 4, E and F). To define the mechanisms mediating the MAIT cell–dependent control of *Klebsiella*, we injected mice with blocking antibodies prior to infection (Fig. 4 G). We found that the MAIT cell protective effect was independent of MR1 but dependent on type I IFN (Fig. 4 G). Accordingly, injection (prior to infection) of αIFNAR blocking antibody or αSiglecH (which reduces type I IFN secretion by plasmacytoid dendritic cells; Blasius et al., 2006) resulted in increased bacterial loads recovered from the lungs of infected mice (Fig. 4 G). Furthermore, the protective MAIT cell function was also lost when we expanded MAIT cells (5-OP-RU+LPS as above) in IFNAR-KO mice as we recovered increased bacterial counts in the lungs of these animals vs. WT controls (Fig. 4 H). Thus, expansion of MAIT cells is sufficient to confer protection from *Klebsiella* infection through a mechanism dependent on type I IFN but independent of MR1.

Our data are in agreement with previous reports in which MAIT cell expansion with 5-OP-RU in combination with TLR ligands or IL-23 provides protection from bacterial infection (Wang et al., 2018, 2019). Similarly, adoptive transfer of pulmonary MAIT cells into immunodeficient mice, protects from infection with influenza or *L*.* longbeachae* (van Wilgenburg et al., 2018; Wang et al., 2018). Interestingly, whereas protection by transferred MAIT cells is dependent on MR1 during *Legionella* infection (Wang et al., 2018), in the case of *Klebsiella* and influenza (van Wilgenburg et al., 2018), MAIT cells are able to exert antimicrobial functions in the absence of MR1. The mechanisms by which MAIT cells promote protection from *Klebsiella* will require further investigation, but it is possible that they contribute to bacterial clearance either by direct secretion of cytolytic molecules or by indirectly controlling the activation and recruitment of other immune cells. For instance, in response to *E. coli*, release of granzymes and granulysin by human MAIT cells mediates control of both cell-associated and extracellular forms of the bacteria (Boulouis et al., 2020). In this setting, granulysin contributes to direct killing of extracellular bacteria, while Granzyme B reduces cell-associated bacteria. Although murine cells do not secrete granulysin, *K. pneumoniae* can survive within macrophages (Cano et al., 2015), thus MAIT cell–derived granzyme could contribute to kill cell-associated bacteria. Added to this, MAIT cell–derived IFN-γ has been shown to be protective during influenza or *L. longbeachae* infections (van Wilgenburg et al., 2018; Wang et al., 2018), and IFN-γ deficient mice are highly susceptible to *Klebsiella* infection (Moore et al., 2002), suggesting further possible mechanisms for anti-bacterial MAIT cell functions.

### Type I IFNs modulate tissue location of MAIT cells during pulmonary *Klebsiella* infection

**Figure 5.**
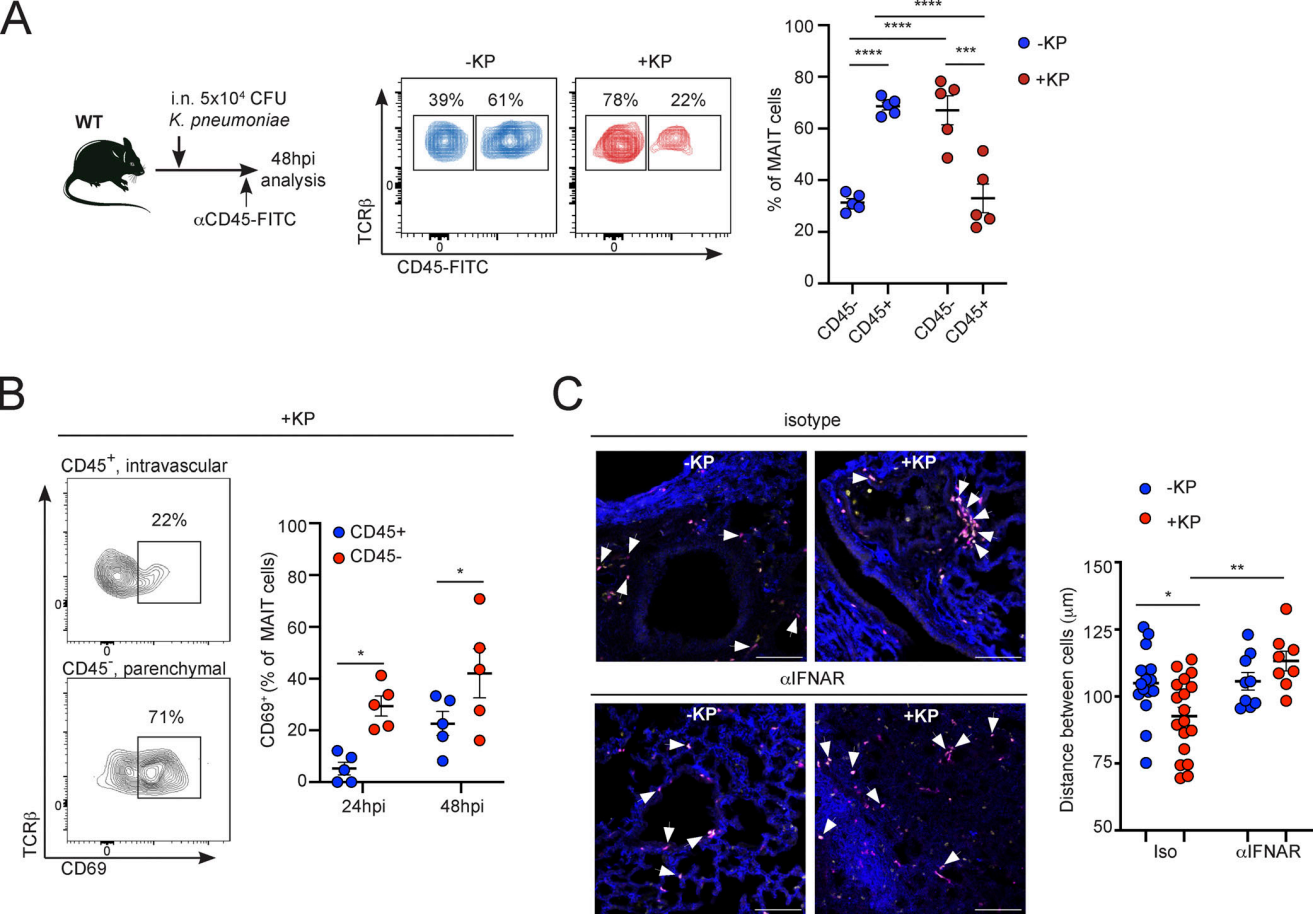
***K. pneumoniae* infection induces MAIT cell relocation in the lung tissue. (A and B)** WT mice were infected with *K. pneumoniae* or received PBS (−KP, control) and were i.v. injected with FITC-labeled αCD45 antibody 3 min prior to tissue collection. **(A)** Representative flow cytometry plots (middle) and quantification (right) of the frequency of pulmonary MAIT cells stained (CD45^+^) or not (CD45^−^) with FITC-labeled αCD45. **(B)** Representative flow cytometry plots (left) and quantification (right) of the frequency of CD69^+^ pulmonary MAIT cells stained (CD45^+^) or not (CD45^−^) with FITC-labeled αCD45. Lines represent mean ± SEM, each dot is a mouse (*n* = 5), and data are pooled from two to three independent experiments. *P < 0.05, ***P < 0.001, ****P < 0.0001, two-way ANOVA with Tukey’s multiple comparisons test. **(C)** Left: Staining of lung tissue (FLASH) for CXCR6-GFP mice (previously injected with 5-OP-RU+LPS to expand the MAIT cell population) showing GFP (pink), CD3 (yellow), and podoplanin (blue). Arrowheads indicate MAIT cells identified as CD3^+^GFP^+^ cells. Mice were infected with *K. pneumoniae* (+KP) or received PBS (−KP) and αIFNAR blocking antibody (bottom) or isotype control (top) prior to infection. Scale bar = 50 μm. Right: Quantification of cell distance evaluated by measuring the spatial distance (μm) between CD3^+^GFP^+^ cells. Each dot represents the average distance (μm) between all CD3^+^GFP^+^ cells per frame (*n* = 9–18 frames per condition) from two independent experiments. *P < 0.05, **P < 0.01, two-way ANOVA with Tukey’s multiple comparisons.

Given that MAIT cells limit bacterial expansion in the lungs and preserve lung pathology during infection, we investigated the effect of bacterial infection in MAIT cell spatial location in the lung (Fig. 5). First, we analyzed the location of MAIT cells in the lung by intravenously injecting fluorescently labeled anti-CD45 antibody (CD45-FITC) 3 min before tissue collection. This approach labels CD45^+^ cells located in circulation and leaves cells in the interstitial tissue and alveoli unlabeled (Salou et al., 2019). Analyses of pulmonary MAIT cells at steady-state demonstrated that around 30% of MAIT cells are located in the lung parenchyma and inaccessible to CD45 labeling. However, the proportion of unlabeled cells significantly increased to around 70% at 48 h after infection, suggesting that cells are relocating into the tissue (Fig. 5 A). Moreover, unlabeled (parenchymal, anti-CD45^−^) MAIT cells showed an increase in expression of the activation marker CD69, in comparison with circulating MAIT cells (intravascular, anti-CD45^+^; Fig. 5 B), suggesting local activation within the tissue.

Next, we investigated how *Klebsiella* infection influenced the location of pulmonary MAIT cells in situ. To do this, we took advantage of CXCR6-GFP mice as MAIT cells (as well as other unconventional T cells) express CXCR6 in the lung and can be identified in the tissues on the basis of GFP expression (Fig. S3, E and F). MAIT cells represent around 5% of the GFP^+^CD3^+^ population in CXCR6-GFP lungs, but after MAIT cell expansion (5-OP-RU+LPS as above) MAIT cells constitute ∼80% of GFP^+^CD3^+^ cells (Fig. S3 F). Thus, we expanded MAIT cells in the lungs of CXCR6-GFP mice and analyzed their tissue distribution by FLASH (fast light-microscopic analysis of antibody-stained whole organs; Messal et al., 2021). This approach enabled us to perform high-resolution immunofluorescence in 3D sections of lungs at steady-state as well as after *Klebsiella* infection. In the absence of infection, MAIT cells (GFP^+^CD3^+^, white arrows) appear widely dispersed along the alveolar parenchyma (in blue, podoplanin^+^ cells). However, 48 h after *Klebsiella* infection, MAIT cells relocate and accumulate in the peri-bronchial spaces (Fig. 5 C). Cell crowding around this region was quantified by calculating the average distance (μm) between all cells appearing in an image (Zepp et al., 2017). Distance between cells was significantly reduced after *Klebsiella* infection, confirming the targeted recruitment of MAIT cells to peri-bronchial spaces induced by *Klebsiella* (Fig. 5 C). Since type I IFNs control the expression of chemokines involved in lung homing of T cells (Padovan et al., 2002; Voillet et al., 2018), we hypothesized that MAIT cell relocation could also depend on type I IFN signaling. Indeed, MAIT cell clustering was prevented by injection of mice with αIFNAR blocking antibody prior to infection (Fig. 5 C). Thus, type I IFNs modulate tissue location of pulmonary MAIT cells during infection.

### Concluding remarks

Our data identify type I IFNs as key drivers of MAIT cell functions during pulmonary infection with *K. pneumoniae*. Type I IFNs act on MAIT cells controlling their activation, effector functions, and induction of a Th1/cytotoxic transcriptional program, ultimately contributing to host protection. Thus, during *Klebsiella* infection, MAIT cells become potent effectors by sensing inflammation independently of cognate antigen. This behavior strongly resembles that of memory CD8^+^ T cells, whose effector functions (Kohlmeier et al., 2010; Soudja et al., 2012) and trafficking (Sung et al., 2012) can be regulated by type I IFNs in an antigen-independent manner. The cytokine-mediated activation of MAIT cells in response to *Klebsiella* contrasts with the MR1-dependent activation during infection with other bacteria such as *F*.* tularensis* or *L. longbeachae* (Meierovics et al., 2013; Wang et al., 2018, 2019). The distinct timeframes of the infections (2 d vs. 2 wk) and the different activation/recruitment of immune cells associated with the specific pathogens may contribute to these differences. Accordingly, it is likely that the timing and (local) concentration of type I IFN delivery accompanying different infections will critically regulate subsequent MAIT cell responses. In keeping with this, IFN-β treatment of human naïve T cells induces several temporal transcriptional waves that regulate the dynamics of T cell activation and differentiation (Sumida et al., 2022). Given the abundance of MAIT cells in humans and their rapid response to inflammatory signals, we propose that type I IFNs may serve as a new molecular target to manipulate MAIT cell functions during infections.

## Materials and methods

### Mice, cells, and chemicals

Mice (WT, IFNAR1-KO, TCRα-KO, MR1-KO on C57BL/6J background; and CXCR6-EGFP “knock-in” in FVB/N background—the latter kindly provided by Adrian Hayday at the Francis Crick Institute, London, UK) were bred under specific pathogen–free conditions at the Francis Crick Institute animal facility. Experiments were carried out using 7–10-wk-old mice with age and gender-matched between genotypes. All experiments have been approved by the Francis Crick Institute and the King’s College London’s Animal Welfare and Ethical Review Body, and UK Home Office under the project license P0BD71419, performed in accordance with the Animals (Scientific Procedures) Act 1986.

Healthy human PBMCs were isolated from leukocyte cones purchased from the NHS Blood and Transplant (UK) and obtained from fully anonymized donors with informed consent. PBMCs were flushed from the leukocyte cones using a syringe and isolated using density gradient centrifugation.

RAW264.7 cells (ATCC TIB-71) were cultured in DMEM (Gibco; 10% FBS, 100 U/ml penicillin, 100 μg/ml streptomycin, and 14.3 μM β-mercaptoethanol) at 37°C in 5% CO_2_.

5-OP-RU was freshly prepared by mixing 5-A-RU and methylglyoxal (Sigma-Aldrich) as previously described (Li et al., 2018).

### Bacterial strains and growth conditions

*K. pneumoniae* (43816 strain, O1K2 serotype) cultures were grown in Luria-Bertani (LB) broth from glycerol stocks at 37°C overnight with shaking at 180 rpm. For the preparation of the inoculum for in vivo infections, bacteria were reinoculated in prewarmed LB medium for 1–2 h culture until reaching OD_600_ = 0.6 (with the estimation that 1 OD_600_ = 7 × 10^8^ CFUs/ml). The bacterial pellet was washed and diluted in phosphate-buffered saline (PBS) for i.n. delivery to mice under isoflurane-induced anesthesia. Bacterial concentration in the inoculum was verified by counting CFUs grown on LB Agar plates.

To construct a *Klebsiella* *ribD* mutant (KPΔ*ribD*), we used λ-red-mediated homologous recombination, as described previously (Hancock et al., 2020). Homologous regions upstream and downstream of *ribD* were amplified via PCR using primers Kp43-*ribD*.2 (5′-GCA​TTG​CCC​TTT​CTG​TTT​C-3′) and Kp43-*ribD*.3 (5′-GGA​ATA​GGA​ACT​AAG​GAG​GAA​GGA​TGG​GTA​GTA​AAG​CG-3′) as well as Kp43-*ribD*.4 (5′-CCT​ACA​CAA​TCG​CTC​AAG​ACT​GCC​TGC​ATT​TAA​CCA​CTG-3′) and Kp43-*ribD*.5 (5′-GCT​TCT​GCA​CCT​TTG​TTA​CC-3′), respectively, and they were sewn together with the kanamycin cassette amplified from pKD4 (Datsenko and Wanner, 2000) using primers cm.3a (5′-TCC​TCC​TTA​GTT​CCT​ATT​CC-3′) and cm.4a (5′-GTC​TTG​AGC​GAT​TGT​GTA​GG-3′). The PCR products were amplified with primers Kp43-*ribD*.2 and Kp43-*ribD*.5, and the PCR fragment was purified with a Qiagen MinElute Kit. Strain Kp43 was transformed with pKOBEG-gent, which encodes the λ phage redγβα operon (Balestrino et al., 2005) and was expressed under the control of the arabinose-inducible pBAD promoter. Briefly, Kp43816 was grown overnight and subcultured 1:100 in 200 ml of LB supplemented with gentamicin and 0.2% arabinose. Cells were grown at 37°C until reaching an OD_600_ of 0.4, cooled on ice, pelleted, resuspended in ice-cold 10% glycerol, and incubated on ice for 1 h. The cells were electroporated with 1 μg three-way-PCR DNA in a 2-mm cuvette with 2.5 kV, 200 Ω, and 25 μF. Transformed cells were selected via plating on LB agar supplemented with kanamycin and riboflavin at 30°C. The replacement of the WT allele by the mutant was confirmed via PCR using primers Kp43-*ribD*.1 (5′-TGC​TTA​ATG​GTA​GCC​AAA​CC-3′) and Kp43-*ribD*.6 (5′-CAG​GAA​CTG​GTA​TTC​GAC​ATC-3′). The pKOBEG-gent plasmid was cured from the mutant strain via overnight growth at 37°C. *ribD* is an essential gene in *K. pneumoniae* and therefore the mutant needs to be grown in the presence of riboflavin (10 ng/ml).

### In vivo inoculations and bacterial infections

For infection experiments, mice were i.n. challenged with 5 × 10^4^ CFUs of *K. pneumoniae* in 30 μl of sterile PBS. For in vivo antibody-mediated blocking experiments, mice were intraperitoneally (i.p.) injected with the following antibodies 24–48 h before infection: αMR1 (150 μg/mouse; clone 26.5; Biolegend, Mouse IgG2a), αIFNAR1 (200 μg/mouse; clone MAR1-5A3; BioXCell, Mouse IgG1), αSiglecH (150 μg/mouse; clone 440c; BioXCell, Rat IgG2b), or their respective isotype controls: Mouse IgG2a (150 μg/mouse; clone MOPC-173; Biolegend), Mouse IgG1 (200 μg/mouse; clone MOPC-21; BioXCell), and Rat IgG2b (150 μg/mouse; clone LTF-2; BioXCell). When indicated, MAIT cell expansion in vivo was performed through a repeated i.n. inoculation (3×) of 5-OP-RU (100 μM) and LPS (17.4 μg/mouse; Invivogen).

Bacterial loads were determined by counting CFUs after plating 100-fold dilution series of tissue homogenates obtained from bacteria-infected mice. Colonies were counted at 24 h.

### Tissue processing, flow cytometry, and MAIT cell sorting

Mice were euthanized by cervical dislocation, and spleen, inguinal lymph nodes, liver, and lungs were harvested. Lungs, inguinal lymph nodes, and liver were finely chopped and digested with 1.5 mg/ml collagenase D (Sigma-Aldrich), 0.1 mg/ml DNase I (ITW Reagents), 0.6 mg/ml NADase (Sigma-Aldrich), and 5% FBS (Thermo Fisher Scientific) in PBS for 60 min at 37°C with gentle shaking. Cells were then passed through a cell strainer (40–70 μm) and washed with sterile PBS. For flow cytometric analysis, cell suspensions were depleted from red blood cells using the hypotonic buffer Tris-based amino-chloride (5 min, room temperature) prior to staining with the antibodies depicted below.

Flow cytometry staining was performed in FACS buffer (1% FBS, 1% BSA, 0.02% sodium azide) using the following antibodies from Biolegend unless specified otherwise: Anti-mouse antibodies: B220 (RA3-6B2), CD11b (M1/70), CD11c (N418), CD27 (LG.3A10), CD28 (37.51), CD69 (H1.2F3), CD25 (PC61), CD44 (IM7), CD137 (17B5), ICOS (7E.17G9; Invitrogen), PD-1 (29F.1A12), TCRβ (H57-597), TCR γ/δ (clone GL3), IL-17A (TC11-18H10.1), IFNγ (XMG1.2), Granzyme B (QA16A02), and pSTAT1(Ser727; A15158B). Anti-human antibodies were as follows: CD14 (HCD14), CD19 (HIB19), CD3ε (HIT3a), CD8α (HIT8a), CD161 (HP-3G10), Vα7.2 (3C10), CD69 (FN50), and Granzyme B (QA16A02). Dead cells were excluded from the analyses using Zombie fixable viability dye (Biolegend). For intracellular staining, cells were fixed and permeabilized using BD Cytofix/Cytoperm Solution kit (BD Biosciences) according to the manufacturer’s instructions. MR1(5-OP-RU), MR1(6-formyl pterin, Ac-6-FP), and CD1d(PBS-57)-loaded tetramers were provided by the National Institutes of Health (NIH) Tetramer Facility. The MR1 tetramer technology was developed jointly by James McCluskey, Jamie Rossjohn, and David Fairlie, and the material was produced by the NIH Tetramer Core Facility as permitted to be distributed by the University of Melbourne. Data were recorded using LSR-II or LSR Fortessa cytometers (BD Biosciences) and analyzed with FlowJo software (TreeStar).

For cell sorting of pulmonary murine MAIT cells, T cells were previously enriched in lung single-cell suspensions using αCD3ε microbeads (Miltenyi Biotec). Murine MAIT cells were sorted as Zombie^−^B220^−^CD11b^−^CD11c^−^TCRβ^+^MR1(5-OP-RU) tetramer^+^ cells.

Human MAIT cells were sorted from PBMCs as Zombie^−^CD14^−^CD19^−^CD3ε^+^CD8α^+^CD161^+^Vα7.2^+^ cells.

### Adoptive transfer

For adoptive transfer experiments, pulmonary MAIT cells (WT or IFNAR-KO) were isolated from the lungs of mice after expansion with 5-OP-RU/LPS as described above. 2–3 × 10^5^ MAIT cells were injected into the tail veins of TCRα-KO recipient mice. To control residual contamination of conventional T cells, mice were i.p. injected with 100 μg each of αCD4 (clone GK1.5; BioXCell, Rat IgG2b) and αCD8 (clone 53-6.7; BioXCell, Rat IgG2a) blocking antibodies on days 2 and 6. To allow full expansion of the MAIT population in the lungs, mice were rested for 14 d prior to infection with *K. pneumoniae*.

For adoptive transfer into IFNAR-KO or MR1-KO mice, 2–4 × 10^5^ MAIT cells were injected into the tail veins of recipient mice. Mice were infected with *Klebsiella* 7 d after transfer.

### Lung histopathological evaluation

Harvested lungs were fixed in 10% neutral buffer formalin before routine histological processing in paraffin wax. 3-μm-thick sections were cut and H&E staining was performed following standard protocols. H&E-stained slides were evaluated concurrently by two board-certified veterinary pathologists (A. Suárez-Bonnet and S.L. Priestnall) blinded to the experimental groups using an BX43 microscope (Olympus). Representative slides were scanned using Panoramic Scan II slide scanner (3D Histech). Presence of airway inflammation (cell infiltration of the airways, bacterial presence within inflamed regions) was examined in the sections. Additional tissue sections were immunohistochemically stained using a rabbit monoclonal antibody anti-F4/80 (clone EPR26545-166; Abcam) to highlight macrophages and semiquantitatively evaluated. Standard histopathological criteria were used for the assignment of the lung injury scores as follows: score 0 (no lesion, 1–4%), score 3 (minimal, 5–9%), score 6 (mild, 10–19%), score 9 (moderate, 20–50%), and score 12 (marked, >51%).

### In vitro stimulation and coculture experiments

For in vitro stimulation experiments, MAIT cells were sorted from the lungs of mice (previously injected with 5-OP-RU+LPS to expand the MAIT cell population). Cells were incubated with IFNα (1 μg/ml; Biolegend) or the supernatant of RAW264.7 macrophages previously exposed to *Klebsiella* (for 2 h).

For coculture experiments with murine cells, T cells from spleen and inguinal lymph nodes (negatively selected using biotin anti-mouse B220 antibody [RA3-6B2] and Dynabeads Biotin Binder [Invitrogen]) were cocultured with RAW264.7 macrophages (1:2 cell ratio) in the presence of live *K. pneumoniae* (multiplicity of infection [MOI] 100) for 3 h. Alternatively, sorted pulmonary MAIT cells (as above) were cocultured with RAW264.7 macrophages (1:4 cell ratio) in the presence of fixed *K. pneumoniae* (100 bacteria per cell) for 18 h. For detection of intracellular cytokines, brefeldin A (Sigma-Aldrich) was added to the final 4 h of the coculture. In some experiments, the following blocking antibodies were added to the cultures: αMR1 (26.5; Biolegend, Mouse IgG2a), αIFNAR1 (MAR1-5A3; BioXCell, Mouse IgG1), αIL-12/23p40 (C17.8; Biolegend, Rat IgG2a), αIL-18 (YIGIF74-1G7; BioXCell, Rat IgG2a); or respective isotype control antibodies: Mouse IgG2a (clone MOPC-173; Biolegend), Mouse IgG1 (clone MOPC-21; BioXCell), Rat IgG2a (clone RTK2758; Biolegend), and Rat IgG2a (clone 2A3; BioXCell).

For in vitro experiments with human cells, CD8^+^ T cells and CD14^+^ monocytes were isolated from PBMCs using αCD8α and αCD14 microbeads (Miltenyi Biotec). Cells were cocultured in the presence of fixed *K. pneumoniae* (100 bacteria per cell) in a 2:1 cell ratio (CD14^+^:CD8^+^ cells) in complete RPMI medium (Gibco; 10% FBS, 100 U/ml penicillin, 100 μg/ml streptomycin, and 14.3 μM β-mercaptoethanol). For detection of intracellular cytokines, brefeldin A (Sigma-Aldrich) was added to the final 4 h of the coculture. In some experiments, αMR1 (1 μg/ml, clone 26.5; Biolegend, Mouse IgG2a) or vaccinia virus B18R protein (5 μg/ml; eBioscience) were added to the cultures.

### RNAseq and data analysis

Human PBMCs (from four different healthy donors) were incubated during 20 h with fixed *K. pneumoniae* (100 bacteria per cell) or, alternatively, with IFNα2A (50 ng/ml; Sigma-Aldrich) or influenza A virus (H3N2, X31 strain, MOI 1). MAIT cells were sorted as Zombie^−^CD14^−^CD19^−^CD3ε^+^CD8α^+^CD161^+^Vα7.2^+^ cells, and RNA was extracted with NucleoSpin RNA XS kit (Macherey-Nagel) following the manufacturer’s instructions. Libraries were generated using NEBNext Ultra II Directional PolyA mRNA kit (New England Biolabs), barcoded, and run on an Illumina NovaSeq 6000 system generating 25 million single-end 75 bp reads per sample. Gene expression was quantified from raw FastQ files in the GRCh38 genome with Ensembl release-95 gene models using the nf-core/rnaseq pipeline (version 3.3; Ewels et al., 2020). Reads were trimmed for adapters with trimgalore (version 0.6.6) and aligned and quantified with RSEM/STAR (version 1.3.1). Data quality was inspected using FastQC (version 0.11.9), Picard (version 2.23.9), and RSeQC (version 3.0.1). Quantified gene expression tables were loaded into R (version 4.1.1) using the tximport package (version 1.20.0; Soneson et al., 2015). DEGs resulting from each treatment, while accounting for between-donor variation, were identified using the DESeq2 package (version 1.32.0; Love et al., 2014). An adjusted P value threshold of 0.01 and log_2_ fold change >0.5 were applied to provide lists of DEGs. Sequencing data have been deposited in the National Center for Biotechnology Information's Gene Expression Omnibus repository under the accession number GSE218601.

### Microscopy

MAIT cells were expanded (with 5-OP-RU/LPS as described above) in CXCR6-EGFP mice which were subsequently infected with *Klebsiella*. Lungs were harvested and fixed overnight in 4% paraformaldehyde (Sigma-Aldrich). Once fixed, antigen retrieval, sample preparation, and staining were performed following the FLASH immunofluorescence protocol (Messal et al., 2021). Samples were stained with rabbit polyclonal αCD3ε antibody (Abcam), αPodoplanin/gp36 (PMab-1; Abcam), and goat polyclonal αGFP (Abcam) antibodies followed by donkey anti-rabbit IgG Alexa Fluor Plus 594 (Invitrogen), donkey anti-rat IgG DyLight 680 (Invitrogen), and donkey anti-goat IgG Alexa Fluor 488 (Invitrogen). Images were acquired by using an inverted Zeiss LSM 710 microscope. Cell clustering was calculated (Zepp et al., 2017) by measuring the average distance (μm) between CD3^+^GFP^+^ cells on at least 9–10 different images per condition by using Fiji/Image J software.

### Statistical analysis and figure preparation

Statistical tests were performed using Prism GraphPad software (version 9.4.0). Comparison between groups was performed using Student’s *t* tests, one-way, two-way ANOVA tests, or Mann-Whitney tests as appropriate unless otherwise stated.

Some figures were drawn using pictures from Servier Medical Art. Servier Medical Art by Servier is licensed under a Creative Commons Attribution 3.0 Unported License (https://creativecommons.org/licenses/by/3.0/).

### Online supplementary material

Fig. S1 shows data related to Fig. 1, including MAIT cell gating strategy and additional controls for αMR1 in vivo. Fig. S2 shows additional RNAseq analyses and data related to Fig. 2, and Fig. S3 shows data related to Fig. 2 (pSTAT1 time-course), Fig. 3 (quantification of MAIT cell responses to WT *Klebsiella* and KPΔ*ribD*), Fig. 4 (phenotyping of MAIT cells after expansion in vivo with LPS+5-OP-RU), and Fig. 5 (characterization of MAIT cells in CXCR6-GFP mice). Tables S1, S2, and S3 include DEGs obtained from RNAseq for human MAIT cells in response to *Klebsiella*, IFNα, or influenza (vs. control).

## Supplementary Material

Table S1 shows the human *Klebsiella*-stimulated MAIT vs. non-stimulated MAITs gene list.

Table S2 shows the human INFα-stimulated MAIT vs. non-stimulated MAITs gene list.

Table S3 shows the human influenza–stimulated MAIT vs. non-stimulated MAITs gene list.

## Data Availability

Sequencing data have been deposited in the National Center for Biotechnology Information's Gene Expression Omnibus repository under the accession number GSE218601. All other data are available in the article itself and its supplementary materials.
